# Locking GTPases covalently in their functional states

**DOI:** 10.1038/ncomms8773

**Published:** 2015-07-16

**Authors:** David Wiegandt, Sophie Vieweg, Frank Hofmann, Daniel Koch, Fu Li, Yao-Wen Wu, Aymelt Itzen, Matthias P. Müller, Roger S. Goody

**Affiliations:** 1 Department of Physical Biochemistry, Max Planck Institute of Molecular Physiology, Otto-Hahn-Strasse 11, 44227 Dortmund, Germany; 2 Chemical Genomics Centre of the Max Planck Society, Otto-Hahn-Strasse 15, 44227 Dortmund, Germany; 3 Center for Integrated Protein Science Munich (CIPSM), Department Chemistry, Technische Universität München, Lichtenbergstrasse 4, 85747 Garching, Germany

## Abstract

GTPases act as key regulators of many cellular processes by switching between active (GTP-bound) and inactive (GDP-bound) states. In many cases, understanding their mode of action has been aided by artificially stabilizing one of these states either by designing mutant proteins or by complexation with non-hydrolysable GTP analogues. Because of inherent disadvantages in these approaches, we have developed acryl-bearing GTP and GDP derivatives that can be covalently linked with strategically placed cysteines within the GTPase of interest. Binding studies with GTPase-interacting proteins and X-ray crystallography analysis demonstrate that the molecular properties of the covalent GTPase–acryl–nucleotide adducts are a faithful reflection of those of the corresponding native states and are advantageously permanently locked in a defined nucleotide (that is active or inactive) state. In a first application, *in vivo* experiments using covalently locked Rab5 variants provide new insights into the mechanism of correct intracellular localization of Rab proteins.

## Abstract (web summary)

The cellular function of small GTPases is regulated by switching between active (GTP-bound) and inactive (GDP-bound) states. Here the authors develop nucleotide analogues that can be covalently linked to GTPases via a strategically placed cysteine residue to lock the target GTPase in defined activation states.

## Graphical abstract

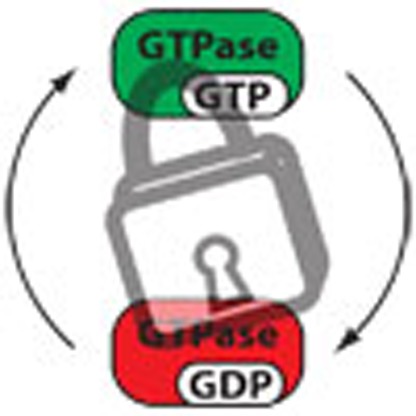

Proteins of the Ras superfamily of small GTPases regulate a variety of cellular processes such as intracellular transport, cell shape and motility, as well as differentiation and growth^1^. For all of these processes, switching between the active GTP-bound and the inactive GDP-bound states is of fundamental importance^2^. The interconversion of these states is brought about by guanine nucleotide exchange factors (GEFs) that catalyse the GDP–GTP exchange and GTPase-activating proteins (GAPs) that accelerate GTP hydrolysis^3^. The cellular and biochemical investigations of the role of GTPases have been substantially furthered by the generation of the proteins in forms corresponding to their active or inactive functional states. This includes the use of non-hydrolysable GTP analogues and the use of amino acid substitutions intended to stabilize the active^4^ or inactive^5^ states of the proteins. Despite numerous successful applications of these strategies, there are situations in which these approaches are inadequate. For example, nucleotide analogues mimicking GDP or GTP may potentially exchange against the endogenous natural nucleotides in *in vivo* experiments, or specific GTPase amino acid substitutions may affect the molecular properties in a more complex and non-predictable manner than intended^6^,^7^. The latter particularly applies to the targeted GDP state, and there is essentially no method available for generating a conformation that is a true analogue of GTPase:GDP complexes without compromising other fundamental properties of the proteins (for example, binding to GEFs)^8^. We reasoned that these problems could be avoided by generating covalent GTPase:nucleotide complexes and thus preventing GDP/GTP displacement by endogenous guanine nucleotides. For this approach to be successful, the covalent adducts would have to adopt exactly the same conformations and maintain identical molecular properties as the native GTPase:nucleotide complexes (with the exception of the desired irreversible nucleotide binding).

We describe here the synthesis of reactive acryl derivatives of guanine nucleotides that are able to react covalently with strategically placed cysteines in GTPases to lock them into their functional states in an irreversible manner. Extensive analysis of biochemical and structural features demonstrate the maintenance of the signalling ability of the modified GTPases. In the case of Rab proteins, we show that the covalent adducts can be used to answer questions concerning the mechanism of localization to specific intracellular membranes.

## Results

### Synthesis of reactive acryl adducts of guanine nucleotides

**Figure 1:**
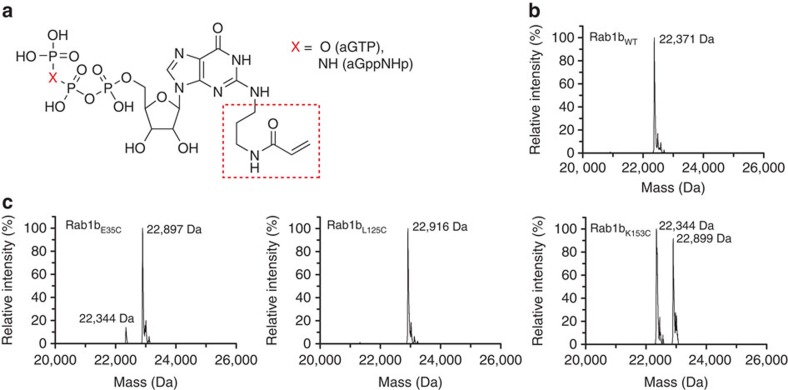
Covalent modification of Rab proteins. (**a**) Structure of acryl–nucleotides synthesized in this publication (GTP and GppNHp—Guanosine 5′-[β,γ-imido]triphosphate). The introduced linker (referred to as acryl-) is highlighted in the red box. (**b**) Rab1b_WT_ (WT-wild type; expected size without modification 22,365 Da) was not modified by aGTP even though it contains three cysteines. (**c**) In contrast, the mutant proteins Rab1b_E35C_, Rab1b_L125C_ and Rab1b_K153C_ (expected sizes without modification 22,339, 22,355 and 22,340 Da, respectively) were all modified to form the covalent aGDP adducts.

The design of a reactive guanine nucleotide derivative that can be covalently linked to a given engineered GTPase needs to fulfil specific requirements. In particular, a position in the guanosine molecule must be found where chemical modifications are compatible with initial high affinity binding into the nucleotide-binding pocket of the GTPase. From our earlier work, we know that modifications at the N2 position of the guanine base are well tolerated by Ras family GTPases, as long as one hydrogen atom remains for an essential interaction with a conserved aspartate residue of the nucleobase binding NKxD motif of the protein^9^. We therefore designed a synthetic strategy to prepare analogues of guanine nucleotides bearing an additional group on the N2 amino function that is able to react covalently with strategically placed cysteine side chains in appropriately mutated proteins. Starting from 2′,3′,5′-triacetyl guanosine, the N2–acryl-GTP (referred to as aGTP) and N2–acryl–GppNHp (referred to as aGppNHp; GppNHp: guanosine 5′-[β,γ-imido]triphosphate) derivatives (Fig. 1) were prepared in a five-step synthesis (see Supplementary Methods and Supplementary Fig. 1 for the synthesis strategy and the analysis of the intermediates and the final products). The acrylamide residue in these derivatives reacts selectively with thiol groups via Michael addition. Advantageously, the moderate electrophilicity of the acryl group (in contrast to many other thiol-reactive reagents) reduces the probability of non-specific reactions with distantly located cysteines.

### Covalent modification of GTPases with reactive nucleotides

In addition to the nucleotide design, appropriate positions for replacement of a natural amino acid by a cysteine residue need to be identified. Obviously, they must be near enough to the acrylamide moiety of the nucleotide derivative to allow reaction to occur, but the position in the GTPase must be chosen so that the formation of the covalent bond does not affect the interaction with partner proteins. In order to test the molecular effects of covalent nucleotide binding to the GTPase, we chose Rab1b as a model protein since we have characterized many of its interaction partners (that is, GEFs, GAPs, GDP-dissociation inhibitor (GDI) and effectors) in biochemical and/or structural detail^10^,^11^,^12^,^13^,^14^. In a structure-guided approach, we identified a number of potential positions for cysteine introduction and prepared a total of 11 mutant proteins (see Rab1b mutants in Supplementary Table 1 and Supplementary Fig. 2). In order to test the reactivity of these positions, GDP was displaced from the Rab1b:GDP complex using an excess of aGTP. EDTA was added for Mg^2+^ complexation in order to increase the rate of GDP dissociation that would otherwise kinetically limit aGTP binding. Electrospray ionization mass spectrometry (ESI-MS) was used to monitor covalent reactions. Covalently coupled Rab1b–aGTP products were found in only three cases, that is, with the E35C, L125C and K153C mutants (Fig. 1). The K153C mutant was not used for further experiments, since the covalent modification was less efficient than with the other two variants (Fig. 1 and Supplementary Table 2).

After 18–23 h at ambient temperature, ESI-MS showed the presence of species with molecular weights corresponding to covalent adducts between Rab1b and aGDP, even though aGTP was used for modification. This suggests that the traces of Mg^2+^ that still prevail despite the presence of the relatively poor Mg^2+^-chelator EDTA are enough to support GTP hydrolysis, and that this occurs on a similar timescale to the covalent reaction. The fact that covalently coupled aGTP formed initially undergoes the GTPase reaction is a first indication that the properties of Rab1b are still intact. In this context it should be noted that all modification reactions were performed under native conditions without protein denaturation being observed.

Since Rab proteins contain essential C-terminal cysteines that are the sites of geranylgeranylation to allow membrane attachment of Rab proteins^15^, the specificity of reaction of the acryl group towards the artificially introduced cysteines needed to be evaluated. In a control experiment performed under identical conditions to those described above, but using wild-type full-length Rab1b (Rab1b_WT_), no covalent adduct was generated despite the presence of these cysteine residues (Fig. 1). This indicates that the reaction occurs specifically at the introduced cysteines at positions E35C or L125C, and that non-specific labelling does not occur at the C terminus, nor at C23 present in Rab1b_WT_.

Extending the labelling experiments to other GTPases, placing a cysteine at the position equivalent to E35 in Rab1b led to covalent interaction with aGTP for all those tested (Rab5A, Rab6A, Rab7A, Rab8A, Ypt7, HRas and Cdc42), albeit at variable rates, but substitution of cysteine at positions corresponding to L125 in Rab1b only led to covalent reaction in the case of Rab7A (Supplementary Table 2; Supplementary Fig. 3a). The lack of reaction in the latter case is surprising, since the three-dimensional (3D) structure at position 125 is highly conserved. It is possible that the linker length could be optimized in further studies, which might lead to more general application of use of this position. Initially position L125_Rab1b_ was thought to be the better choice for modification than position E35_Rab1b_, since the latter is at the beginning of the effector binding region of switch I, and could thus interfere with effector and other interactions. However, results presented below suggest that this is not the case for the Rab interactions tested.

### Protein–protein interactions of Rab1b–aGTP/aGDP adducts

**Figure 2:**
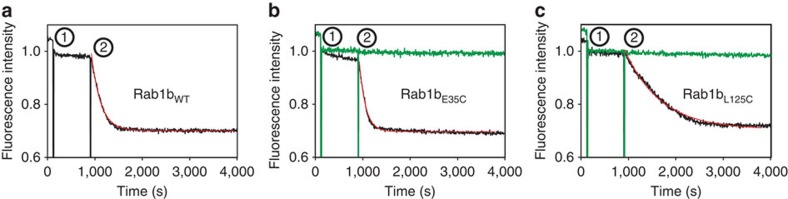
GEF-catalysed nucleotide exchange. (**a**) Rab1b_WT_, (**b**) Rab1_E35C_ and (**c**) Rab1b_L125C_ (black traces) non-covalently bound to GDP were mixed with an excess of GppNHp (Guanosine 5′-[β,γ-imido]triphosphate, step 1) and catalytic amounts of DrrA_340-533_ (DrrA-GEF, step 2). The nucleotide exchange reaction in step 2 was fitted with a single exponential equation yielding observed rate contants of 5.1 × 10^−3^ s^−1^ (Rab1_WT_), 7.5 × 10^−3^ s^−1^ (Rab1_E35C_) and 1.3 × 10^−3^ s^−1^ (Rab1_L125C_). In contrast to the non-covalently nucleotide-bound proteins, both Rab1 mutants containing the covalently bound nucleotides (green traces) did not show any nucleotide exchange upon addition of DrrA-GEF.

Another requirement for the use of the covalent adducts to answer mechanistic or cell biological questions is that their interactions with partner proteins are native like. We therefore tested such interactions for Rab proteins, beginning with GEF-catalysed GDP exchange experiments. First, we validated that the cysteine substitutions did not interfere with the intrinsic nucleotide-binding properties of Rab1b. Incubation of the GDP form of Rab1b_WT_, Rab1b_E35C_ and Rab1b_L125C_ with excess GppNHp in the presence of Mg^2+^ ions did not lead to a significant change of the intrinsic GDP-dissociation rate in the case of Rab1b_WT_ and Rab1b_L125C_, whereas Rab1b_E35C_ showed slightly weakened nucleotide binding as monitored using protein fluorescence as a signal^16^ (shown in step 1, Fig. 2). We then analysed the GEF-catalysed GDP-displacement reaction using the same assay in order to test whether Rab1b modified covalently with aGDP is still a GEF substrate. As expected, the exchange rate was increased dramatically by addition of catalytic amounts of the GEF domain of the Legionella protein DrrA (referred to as DrrA-GEF)^14^ for Rab1b_WT_, Rab1b_L125C_ and Rab1b_E35C_ with small variations of the observed rates (Fig. 2, step 2). In stark contrast, no reaction was observed with the covalent aGDP-modified GTPases Rab1b_E35C_–aGDP and Rab1b_L125C_–aGDP, thus showing that no GEF-mediated displacement of the covalently bound nucleotide occurred, as expected.

The interaction with GEFs is expected to be quite weak for GTPases with covalently attached nucleotides, since the basis of the mechanism of GEF action is that they interact weakly (*K*_d_ values in the range of 10–100 μM) with nucleotide-bound states of the proteins, but very strongly (*K*_d_ values in the nM to pM range) with the nucleotide-free proteins^17^. In keeping with this, full-length DrrA forms a quantitative complex with Rab1b after mixing with Rab1b:GDP but not with Rab1b_L125C_–aGDP as shown by gel filtration chromatography (Supplementary Fig. 5a,b). This observation is in accordance with the idea that Rab1b_L125C_–aGDP maintains its nucleotide state permanently, therefore binding only weakly to the GEF molecule.

Evidence that an interaction of the GDP-locked protein with GEFs does indeed occur was provided by experiments on the effect of alkaline phosphatase on Rab1b_L125C_–aGDP. Expectedly, incubation of Rab1b–aGDP with alkaline phosphatase did not result in removal of the nucleotide phosphate groups, suggesting that they are not sterically available to the enzyme. On the basis of extensive knowledge of GTPase–nucleotide structures and the very tight association between the protein and the phosphate groups, this is not surprising^3^. However, addition of a stoichiometric amount of DrrA-GEF resulted in alkaline phosphatase-dependent removal of the phosphate groups to produce the guanosine derivative (Rab1b_L125C_–aGuanosine, Supplementary Fig. 6a). This suggests that interaction of Rab1b_L125C_–aGDP with DrrA-GEF opens the nucleotide-binding site sufficiently for alkaline phosphatase to hydrolytically cleave the exposed phosphate groups. This is in keeping with the known dramatic nucleotide exposing effect of DrrA on the structure of the loop regions of Rab1 (ref. 14; Supplementary Fig. 6b). Interestingly, DrrA was present as a stable complex with Rab1b_L125C_–aGuanosine at the end of the reaction, and this could not be disrupted by high concentrations of GDP as is seen for native Rab:GEF complexes (Supplementary Fig. 6c,d). Since many GEFs stabilize the intermediate nucleotide-free state of Rab proteins by substituting the missing interactions between the proteins' nucleotide-binding pockets and the phosphates^3^, the aGuanosine locked form might represent a fair analogue of this intermediate, explaining the tight association of the DrrA:Rab1b–aGuanosine complex even in the presence of a high excess of GDP. Thus, although GTPases only have a very modest affinity for guanosine (*K*_d_ ∼100 μM for H-Ras^18^), in combination with the high local concentration of the guanosine base with respect to its interacting residues this is enough to prevent the binding of GDP at a concentration of 100 μM despite the picomolar affinity of GDP to Rab1b in the absence of covalently bound nucleotide. This could not yet be tested for the E35C-linked adduct of Rab1b, since treatment of Rab1b_E35C_–aGDP with alkaline phosphatase in the presence of DrrA-GEF did not lead to removal of the phosphate groups of GDP (Supplementary Fig 6a). This is presumably because the large conformational change occurring on interaction of Rab1 with DrrA involves a dramatic movement of the switch I region^14^ which is inhibited because of the immobilization of switch I by the covalent linkage (Supplementary Fig. 6b).

**Figure 3:**
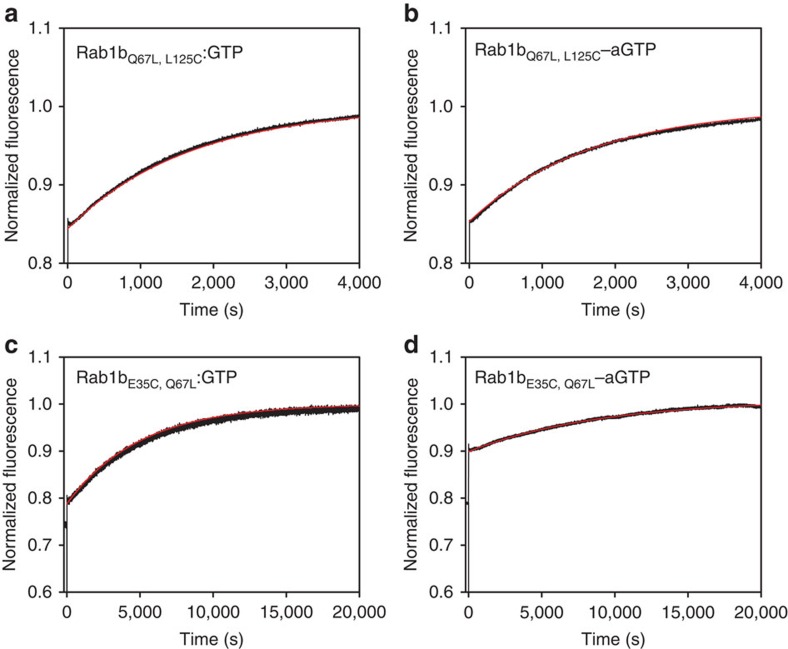
GAP-catalysed GTP hydrolysis. 4 μM (**a**) Rab1_Q67L, L125C_:GTP, (**b**) Rab1_Q67L, L125C_–aGTP, (**c**) Rab1_E35C, Q67L_:GTP and (**d**) Rab1_E35C, Q67L_–aGTP were incubated with 0.4 μM TBC1D20_14-305_. The resulting progress curves (black) were fitted using a single exponential equation (red line) and yielded observed rate constants of 6.0 × 10^−4^ s^−1^ (Rab1_Q67L, L125C_:GTP), 5.0 × 10^−4^ s^−1^ (Rab1_Q67L, L125C_–aGTP), 2.0 × 10^−4^ s^−1^ (Rab1_E35C, Q67L_:GTP) and 1.1 × 10^−4^ s^−1^ (Rab1_E35C, Q67L_–aGTP).

The second key interaction in the regulation of G-protein activity is the GTP-hydrolysis reaction catalysed by GAPs. In a first control experiment it was shown that the E35C and L125C mutations in Rab1b do not have an effect on the interaction with the GAP TBC1D20 (Supplementary Fig. 7). In order to prevent the intrinsic GTP-hydrolysis reaction, we generated so-called hydrolysis-resistant variants (containing the Q67L mutation) of Rab1b_L125C_–aGTP and examined the GAP activation of hydrolysis of the covalent adduct. In Rab proteins, the removal of the essential glutamine only has a modest effect on the GAP-catalysed reaction, since a glutamine from cellular Rab–GAPs plays an essential role in the GTPase reaction, in contrast with the situation with most other GAP:GTPase interactions^10^,^19^. Thus, Rab1b_Q67L,L125C_–aGTP could be generated quantitatively, and after addition of the Rab1b–GAP TBC1D20, the increase in protein fluorescence indicated that GTP hydrolysis occurred^16^. The rate of this TBC1D20 stimulated reaction was similar to that of Rab1b_Q67L,L125C_:GTP (Fig. 3a,b). Rab1b_E35C_–aGTP was also a substrate for TBC1D20 with a slightly reduced hydrolysis rate compared with the non-crosslinked protein (Fig. 3c,d).

To fulfil their biological role, Rab proteins need to be modified by post-translational addition of one, or in most cases two geranylgeranyl groups to their C terminus. To be prenylated by RabGGTase (Rab geranylgeranyltransferase), GDP-bound Rab proteins require simultaneous binding to REP (Rab escort protein)^20^,^21^. We therefore tested whether the covalently modified Rab variants could be prenylated by RabGGTase in the presence of REP *in vitro*. Rab1b_L125C_–aGDP was prenylated at the same rate as Rab1b_L125C_:GDP using NBD-farnesyl pyrophosphate (3,7,11-trimethyl-12-(7-nitro-benzo[1,2,5]oxadiazo-4-ylamino)-dodeca-2,6,10-trien-1 pyrophosphate; NBD-FPP) as the lipid substrate (Supplementary Fig. 8). We therefore conclude that the interaction with both REP and RabGGTase was unaffected by the covalent modification. In a similar experiment, Rab1b_E35C_–aGDP could also be prenylated (Supplementary Fig. 8).

Another key interaction of Rab proteins is that with the protein known as GDI (GDP-dissociation inhibitor)^22^,^23^. This protein is structurally related to REP^24^,^25^, and shares the property of solubilization of prenylated Rab proteins. In contrast to REP, GDI only interacts strongly with GDP-bound Rabs in their prenylated form^26^. We therefore generated a Rab1b derivative containing a fluorescent C-terminally located lipid moiety (NBD-farnesyl), which has been used previously to assess Rab–GDI interactions^27^. Rab1b–NBD-farnesyl displays an increase in fluorescence intensity on interacting with GDI that was used to monitor binding^12^. A similar increase was seen with NBD-farnesyl-modified Rab1b_L125C_–aGDP. Kinetic experiments using the stopped flow method showed that both the association and dissociation reactions were indistinguishable for NBD-farnesyl-labelled Rab1b_L125C_:GDP and Rab1b_L125C_–aGDP, with the overall *K*_D_ values being 4.6 and 4.0 nM, respectively (Supplementary Fig. 9). We conclude from this that the interaction of Rab1b with GDI is unaffected by the presence of the covalently bound nucleotide, in this case meaning that the GDP (inactive) state of the GTPase is faithfully preserved in the covalent adduct.

**Figure 4:**
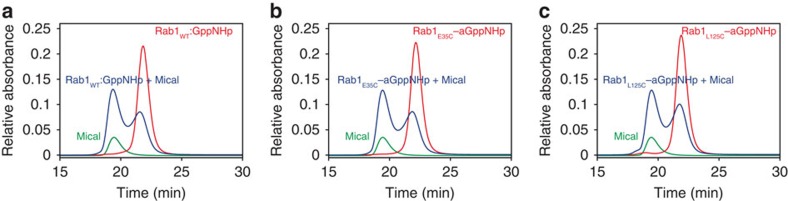
Effector binding of covalently modified Rab proteins. Gel filtration experiments were performed to test for the interaction of the effector protein Mical with Rab1_WT_:GppNHp (**a**) and the covalently locked variants Rab1_E35C_–aGppNHp (**b**) and Rab1_L125C_–aGppNHp (**c**).

Like other GTPases, Rab proteins interact in a GTP-dependent manner with so-called effector proteins. We tested the interaction of the covalent Rab–nucleotide adducts with the Rab1b effector Mical^28^. Analytical gel filtration chromatography showed that complexes were formed between the effector and Rab1b_WT_:GppNHp, Rab1b_E35C_–aGppNHp, or Rab1b_L125C_–aGppNHp (Fig. 4). Similar experiments were performed with Rab1_E35C_ and Rab1_L125C_ non-covalently bound to GppNHp (Supplementary Fig. 10) to exclude effects of the mutations on effector binding. These results demonstrated that the presence of the covalent adduct in the triphosphate form can generate the correct conformation of the protein for effector binding.

In summary, the Rab1 mutants E35C and L125C retain nucleotide and protein binding characteristics compared with those of the wild-type protein. Also, the covalent aGuanosine, aGDP, aGTP and aGppNHp adducts thereof show the expected interactions with GEFs, GAPs, GDI, REP, GGTase II and effectors and thus represent native-like GTPase states.

### 3D structures of covalent adducts of Ypt7

The results described so far indicate that the conformations and properties of Rab1b are retained in a nucleotide-dependent manner in the covalent adducts with aGDP, aGTP or aGppNHp. To further support these conclusions, we aimed at determining the 3D structures of such adducts. Attempts to crystallize adducts with Rab1b were not successful. We did, however, succeed in crystallizing the yeast Rab protein Ypt7 (ref. 29) as the Q35C (analogous to Rab1b_E35C_) mutant of this protein modified covalently with aGDP, quantitatively producing Ypt7_Q35C_–aGDP (Supplementary Fig. 4). In order to determine the structure of the GTP-bound state, we introduced two further mutations in addition to the cysteine at position 35: one of these was Q68L, a position equivalent to the GTP hydrolysis-deficient Rab1_Q67L_ variant. However, Ypt7_Q68L_ hydrolysed GTP faster than Rab1_Q67L_ and we reasoned based on structure and amino acid sequence comparisons that the residue K38_Ypt7_ (I37 in Rab1) close to the γ-phosphate might play a significant role. We therefore introduced a further change at this position (K38I). Using this Ypt7 variant, we were able to produce a covalently linked aGTP adduct that was stable enough to allow crystallization within 12 h at 4 °C and allowed structure determination. In addition to this, we were able to determine the structure of the covalent adduct of the Ypt7_Q35C_ mutant with aGppNHp (for data collection and refinement statistics for Ypt7_Q35C_–aGDP, Ypt7_Q35C_–aGppNHp, and Ypt7_Q35C, K38I, Q68L_–aGTP see Table 1).

**Figure 5:**
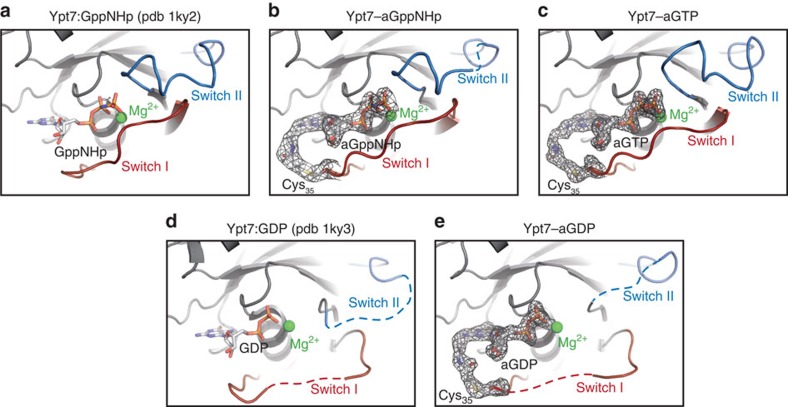
Structures of covalently modified Ypt7. Detailed views and comparison of the nucleotide-binding regions of the previously published Ypt7:GppNHp (**a**) with covalently modified Ypt7–aGppNHp (chain A) (**b**) and Ypt7–aGTP (**c**) as well as the previously published Ypt7:GDP (**d**) and the covalently modified Ypt7–aGDP (**e**). The switch I and II regions are shown in red and blue, respectively, the 2*F*_o_−*F*_c_ electron density (black mesh) around the modified Cys, the linker and the nucleotide is depicted at 1*σ*.

Structural comparison of these adducts with Ypt7:GDP and Ypt7:GppNHp^29^ demonstrates that the covalent linker provides sufficient flexibility to allow the covalently locked proteins to adopt similar conformations of the nucleotide-binding pocket and in particular of the switch I region of Ypt7_Q35C_–aGDP, Ypt7_Q35C_–aGppNHp and Ypt7_Q35C,K38I,Q68L_–aGTP compared with the non-covalently nucleotide-bound proteins (Fig. 5; Supplementary Fig. 11a–c). Despite this flexibility, the electron density around the nucleotide and the linker was well defined in Ypt7_Q35C_–aGDP and Ypt7_Q35C,K38I,Q68L_–aGTP, whereas in Ypt7_Q35C_–aGppNHp this was only true for two (chains A and B) out of four copies within the asymmetric unit. Exemplified *F*_o_−*F*_c_ omit electron density maps and corresponding stereo images are shown in Supplementary Fig. 12a–c and d–f, respectively. In conclusion, the structures of Ypt7 modified with aGDP, aGTP or aGppNHp are very similar to the non-covalent Ypt7:nucleotide complexes, demonstrating that the structural properties of GTPases are maintained in covalently modified proteins.

### Testing the mechanism of Rab localization

One of the essential properties of Rab proteins is their ability to locate to the correct membrane in the cell. This property must be encoded in the structure of each Rab protein, and while it was originally concluded that this information is contained in the hypervariable C-terminal sequence^30^, more recent evidence suggests that although this sequence can be involved in targeting, it is not the dominant factor^31^,^32^. In our own work, we have investigated and developed the hypothesis that the most important single factor is the interaction with GEFs^26^,^33^. According to this model, a GEF that is localized to a specific membrane catalyses displacement of GDP by GTP and thereby recruits its cognate Rab at this membrane, and by doing so prevents dissociation of Rab from this membrane as a complex with GDI because of the low affinity of GDI for Rab:GTP (Supplementary Fig. 13). Recent experiments showing that mislocalized GEFs can recruit their cognate Rabs to the membrane of their mislocalization provide strong support for the hypothesis^33^. Nevertheless, this question is still discussed controversially, and further evidence is required to resolve the issue.

The acryl–nucleotides open up the possibility of testing the mechanism of Rab targeting in a different manner. If the hypothesis that localization is driven by GEF activity at a specific membrane is correct, specific localization should not be seen for covalently locked Rab–aGDP or Rab–aGTP (or Rab–aGppNHp). If, on the contrary, the localization were only dependent on the nucleotide-bound state, the locked Rab proteins would still localize correctly in the covalently locked GTP state.

**Figure 6:**
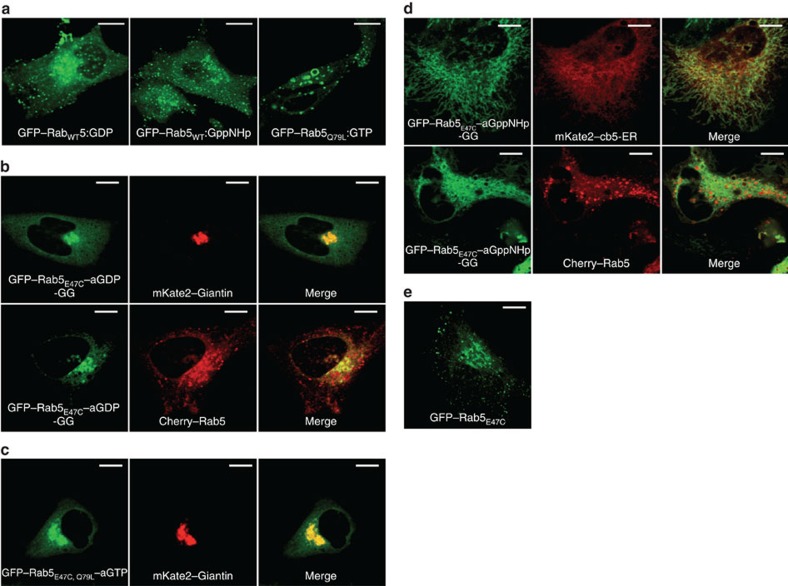
Covalently locked Rab5 does not localize to endosomes. (**a**) GFP–Rab5_WT_ non-covalently bound to GDP (left) or GppNHp (middle) and GFP–Rab5_Q79L_ non-covalently bound to GTP (right) were microinjected into cells. All constructs show a localization resembling that expected for early endosomes. While Rab5_Q79L_ induces formation of enlarged endosomes as previously reported^34^, this is not the case for Rab5_WT_ independently of the nucleotide-bound state (that is, GDP or GppNHp) before microinjection. (**b**) GFP–Rab5_E47C_–aGDP microinjected into cells shows strong localization to the Golgi apparatus instead of endosomes as indicated by co-staining with Giantin and Cherry-Rab5_WT_ (Cherry-Rab5_WT_ was used as a marker of correct intracellular targeting of Rab5 to endosomes). (**c**) GFP–Rab5_E47C, Q79L_–aGTP microinjected into cells is localized to the Golgi apparatus, similar to Rab5–aGDP. This is presumably due to the low remaining hydrolysis activity of Rab5_Q79L_, resulting in the inactive aGDP-bound conformation within the time frame of the experiment. (**d**) GFP–Rab5_E47C_–aGppNHp microinjected into the cell shows a distribution over endomembranous structures throughout the cell with a preference for the endoplasmic reticulum (upper panel; cb5, cytochrome b5), but not specifically to endosomes (lower panel). (**e**) Cells transfected with eGFP-Rab5_E47C_ show a similar localization pattern of Rab5_E47C_ compared with Rab5_WT_. This clearly shows that the E47C mutation alone does not affect intracellular localization (scale bars: 10 μm).

The hypotheses were tested using Rab5, which localizes to early endosomes and produces a strong phenotype with enlarged endosomes in the hydrolysis-deficient Q79L mutant (usually referred to as the constitutively active form)^34^. Exemplified microinjection experiments of Rab5 non-covalently bound to GTP, GDP or GppNHp clearly show this localization to endosomes (Fig. 6a). Similar experiments using Rab5–aGDP showed a strong mislocalization to the Golgi apparatus, while Rab5–aGppNHp showed a mostly cytosolic localization (Supplementary Fig. 14a). Since the active state of Rab proteins is known to be a poor substrate for the prenylation machinery of the cell^21^, we suspected that the latter behaviour was due to lack of prenylation of the Rab5 locked in the active state. The experiments were therefore repeated after prenylation of Rab5 *in vitro* (*in vitro* geranylgeranylated proteins are indicated by the suffix –GG; for mass spectra of Rab5 showing the nucleotide modification and the *in vitro* prenylation, see Supplementary Fig. 14b). In these experiments, both Rab5-aGDP-GG (Fig. 6b) and Rab5_Q79L_–aGTP (Fig. 6c) were not recruited to early endosomes, but again localized to the Golgi apparatus. Significantly, Rab5–aGppNHp–GG was distributed to endomembraneous structures throughout the cell and showed some colocalization with an endoplasmic reticulum marker (Fig. 6d), but did not show the preferential localization to early endosomes typical of correct Rab5 targeting. In order to exclude that the E47C mutation alone led to mistargeting of Rab5, we transfected cells with eGFP-Rab5_E47C_ (Fig. 6e). These experiments clearly showed WT-like localization of the mutant.

Since neither Rab5 locked in the inactive (Rab5–aGDP) nor Rab5 locked in the active (Rab5–aGppNHp and Rab5_Q79L_–aGTP) state localized correctly in these experiments, our results support an important function of GEF-proteins and their catalysis of nucleotide exchange rather than the mere nucleotide state of a Rab protein for targeting to a certain membrane within the cell. However, even though we have shown that the covalent adducts of Rab1 behave in a native-like manner in all aspects tested, we cannot exclude with certainty that hitherto unrecognized effects on the properties of Rab5 might arise from the use of the covalent adducts and thus possibly contribute to the mislocalization observed in our experiments. Further work will be needed to clarify this issue.

## Discussion

The ability to generate GTPases locked irreversibly in either of their two functional states or effectively in their nucleotide-free state raises the possibility of performing experiments in the cellular context that were not previously possible. We have utilized acryl–nucleotides (aGDP, aGTP and aGppNHp) to produce covalently locked Rab proteins that have been equipped with strategically placed cysteines at the beginning of the switch I region or in the guanine base binding NKxD motif. Thorough biochemical characterizations of Rab1b–aGDP, Rab1b–aGTP, Rab1b–aGppNHp and Rab1b–aGuanosine demonstrated that these covalent adducts behaved as expected with GEFs, GAPs, effectors, GDI, REP, and RabGGTase and advantageously retained their nucleotide states. Rab1b was chosen as a model protein since it is the only Rab protein for which the full repertoire of biochemically characterized interaction partners has been established, thus permitting the investigation of the full scope of binding events on a single GTPase. Additionally, covalent GTPase–nucleotide adducts also maintained the native structures and conformations, as demonstrated by the X-ray crystal structures of Ypt7_Q35C_–GDP, Ypt7_Q35C_–aGppNHp and Ypt7_Q35C,K38I,Q68L_–aGTP. Consequently, modifications of small GTPases at positions equivalent to E35 and L125 of Rab1b permit the generation of covalently linked GTPase–acryl–nucleotide adducts that behave identically to the wild-type proteins and therefore allow the elucidation of molecular and cellular processes in which truly guanosine, GDP or GTP-locked states are required.

As an example for an *in vivo* application, we have addressed the mechanism of correct intracellular localization of Rab proteins, which has been a matter of debate for over 20 years, and the factors required for this process are still not completely understood. Recent results favour a decisive role of specifically localized GEFs in targeting of Rab proteins to specific membranes or membrane domains^33^,^35^, although there is evidence that there are possibly additional factors and events involved in this complex but crucial process^36^,^37^. Microinjection of Rab5 locked covalently in the active or inactive state was performed in the present work and the results obtained were in keeping with the idea that for this Rab protein, the process of nucleotide exchange at a certain membrane is a crucial step in correct intracellular localization. The mere presence of Rab5 irreversibly locked in the GTP-bound form in the cell did not lead to correct localization. In the case of the Rab proteins, the importance of the GEF reaction for correct localization might be regarded as limiting the usefulness of the approach we have developed, since Rab GTPases covalently locked in the active state cannot be targeted correctly, at least in the case of Rab5. However, the GEF-driven localization mechanism does not, to the best of our knowledge, apply to all GTPases, so that different types of experiments concerning the roles of the individual nucleotide states can be imagined in such cases. In all cases, it will be of interest to determine the role of nucleotide states on localization and localization dynamics, but of course attention must be paid to the possibility of unforeseen artifacts arising from the approach of using covalently locked GTPases.

Additionally, a number of other possible applications of locking GTPases in their active or inactive states can be envisaged. There are several known examples of GTPases that interact with each other and that are difficult to characterize *in vitro* or *in vivo* because of the difficulty of defining the nucleotide occupancy of each GTPase. Similarly, there are known processes that involve the concerted or sequential action of several GTPases where it would be advantageous to be able to define the nucleotide state of the individual proteins unequivocally. The use of covalently locked states offers a potential solution to some of these issues, for example in structure determination of complexes containing more than one GTPase, in the study of reconstituted systems *in vitro* and in suitable cases for *in cellulo* investigations. In addition to small GTPases, similar approaches will be applicable to research into other classes of GTP/GDP-binding proteins such as heterotrimeric G-proteins. Extension of the principle to ATP-utilizing proteins such as motor proteins or cassette (ABC) transporters^38^ might be an interesting aspect, in particular when intermediate states in enzymatic reactions or certain states of the enzymes in their cellular context are investigated structurally or functionally. Intermediate states are often difficult to investigate due to their short-lived nature as well as for enzymes having high *K*_M_ values for their respective nucleotide substrates, while experiments in cellular contexts suffer from the possibilities of nucleotide exchange with endogenous nucleotides, but these difficulties could be overcome by the use of covalently bound nucleotide analogues. In the case of ATP-dependent enzymes, it is likely that the N6 position will be a suitable site of modification in many cases.

Further development of the substances used in this publication will include optimization of the length and nature of the linker and the reactivity of the electrophilic group used for specific purposes. Extension to other GTPases will require reassessment of the position of introduction of the cysteine group, which should be possibly based on the large number of available structural models of these proteins. A more radical departure from the approach we have described would include the use of a biorthogonal chemical approach (for example, the use of click chemistry) to target specific GTPases or other nucleotide-binding proteins in cells to lock them in their functional states.

## Methods

### Protein expression and purification

All small GTPases were expressed in *Escherichia coli* BL21(DE3) (Cdc42_1-188_ S30C in *E. coli* BL21-CodonPlus (DE3)-RIL). Protein expression overnight at 20 °C was induced at *A*_600 nm_ of 0.6–0.8 by addition of 0.5 mM IPTG (isopropyl-β-D-thiogalactoside; KRas D30C: 0.1 mM IPTG and 37 °C).

For purification, a combination of Ni^2+^ affinity chromatography and (in case of Maltose-binding protein (MBP)-tagged proteins: pMAL and pOPINM plasmids) amylose-affinity chromatography was used. Hexahistidine and MBP tags were removed by protease cleavage followed by a second round of Ni^2+^- or amylose-affinity chromatography, respectively. Finally, all proteins were purified by gel filtration chromatography using HiLoad 16/60 Superdex 75 prep grade columns (GE Healthcare, see Supplementary Table 1 for the respective buffers).

Other proteins were expressed and purified as described earlier (DrrA_340-533_ (referred to as DrrA-GEF) and full-length DrrA^14^, TBC1D20_14-305_ (ref. 10), REP1 (ref. 39), RabGGTase I (ref. 39), GDI-I (ref. 14), KRas D30C^40^ (with additional gel filtration as described above), Mical-3_1841-1990_ (ref. 11), LidA_201-583_ (ref. 13)).

### Covalent modification of proteins

The small GTPases were incubated at a concentration of 50 μM in the presence of 7.5–10 equiv. of the corresponding acryl–nucleotide at 25 °C. In order to increase the rate of nucleotide exchange, a 2–5 fold molar excess of EDTA over MgCl_2_ was added. The completeness of the reaction was controlled by ESI-MS after ∼20 h.

The rate of conversion of the different GTPases estimated from mass spectra (Supplementary Figs 2 and 3) is summarized in Supplementary Table 2. The mutant proteins Rab1b G18C, Rab1b Y33C, Rab1b D89C, Rab1b T91C, Rab1b D92C, Rab1b K122C, Rab1b K128C and Rab1b N154C (Supplementary Fig. 2) could not be modified.

### Guanine nucleotide exchange factor assays

The (GEF-catalysed) nucleotide exchange was monitored via tryptophan fluorescence (emission at 348 nm and excitation at 297 nm) at 25 °C in a FluoroMax-3 spectrofluorometer (Horiba Jobin Yvon) in 20 mM Hepes pH 7.5, 50 mM NaCl, 2 mM Dithioerythritol (DTE), 1 mM MgCl_2_. 2 μM Rab protein were mixed with 50 μM GppNHp (step 1 in Fig. 2) and subsequently with 20 nM DrrA-GEF(step 2 in Fig. 2).

### GTPase-activating protein assays

Rab protein (4 μM) preloaded with GTP or covalently modified with aGTP were mixed with 0.04–0.4 μM TBC1D20. Similarly to the GEF assays, the GAP-catalysed GTP hydrolysis was measured via change in tryptophane fluorescence (excitation 297 nm, emission 348 nm, 25 °C, FluoroMax-3 spectrofluorometer (Horiba Jobin Yvon)).

### Analytical gel filtration

In order to assess the complex formation of Rab proteins with their effector proteins, analytical gel filtration experiments were performed. In each case, 3.9 nmol of the respective Rab protein and 3.4 nmol Mical were either mixed or injected separately in a total volume of 30 μl and subsequently analysed at a flow rate of 0.5 ml min^−1^ on a Superdex 75 10/300 GL column (GE healthcare) in 20 mM Hepes pH 7.5, 50 mM NaCl, 2 mM DTE and 2 mM MgCl_2_. The eluted fractions were analysed via absorption at 280 nm.

Similarly, complex formation of Rab proteins with DrrA-GEF (1.75 nmol of each protein were mixed in a volume of 120 μl) was tested in 20 mM Hepes pH 7.5, 50 mM NaCl, 1 mM tris(2-carboxyethyl)phosphine (TCEP), 1 mM MgCl_2_ with and without 100 μM GDP. Due to the strong absorption of the GDP-containing buffer at 280 nm, the absorption was measured at 295 nm for all experiments containing GDP in the buffer.

### Prenylation of Rab proteins

A volume of 150 μl sample containing 6 μM REP1, 4.7 μM GGTase II, 50 μM NBD-FPP and either 4 μM Rab1b_L125C_–aGDP or 4 μM Rab1b_L125C_:GDP were incubated at 25 °C and 300 r.p.m. in a Thermomixer (Eppendorf AG). At several time points, 10 μl samples were directly diluted in 4 × SDS sample buffer and heated to 90 °C in order to quench the reaction and subsequently analysed by SDS-polyacrylamide gel electrophoresis and in gel fluorescence (excitation 473 nm and emission 510 nm). The programme AIDA Image Analyzer (raytest Isotopenmessgeraete GmbH) was used to quantify in gel fluorescence.

Kinetics of prenylation were measured as described previously^41^. Shortly, 1 μM Rab:REP complex were incubated with 400 nM RabGGTase at 37 °C for 5 min before addition of NBD-FPP. The change of fluorescence was monitored in a fluoromax-3 spectrofluorometer (excitation 479 nm and emission 520 nm) at 37 °C (buffer 20 mM Hepes pH 7.5, 50 mM NaCl, 2 mM DTE and 2 mM MgCl_2_).

### Interaction of prenylated Rab proteins with GDI

NBD-farnesylated Rab1b_L125C_–CVIL:GDP and Rab1b_L125C_–CVIL–aGDP (containing the artificially introduced CaaX-box CVIL at their C termini) were produced by incubating GGTase I, the Rab protein and NBD-FPP (0.5:1:5) for 2 h at 25°C and confirming the completeness of modification by ESI-MS^27^. Observed rate constants of interaction of the prenylated Rab proteins and GDI were measured at concentrations of 100 nM of the respective Rab protein and 100 nM–1 μM of GDI at 25 °C in a stopped flow instrument (SX20, Applied Photophysics Ltd.) via NBD fluorescence (excitation 437 nm and emission filter above 530 nm). Resulting progress curves were fitted using a single exponential equation and observed rate constants were plotted against the GDI concentration to yield the association rate constant (*k*_on_). For determination of the dissociation rate constant (*k*_off_) the complex was preformed at a concentration of 100 nM and mixed 1:1 with 2 μM of the effector protein LidA^13^ in a stopped flow instrument. Displacement of GDI by LidA was measured via NBD fluorescence and the resulting progress curve was fitted single exponentially to yield the *k*_off_.

### Crystallization and structure determination

The proteins were covalently modified with the corresponding acryl-nucleotides as described above in the section on covalent modification of proteins (see mass spectra in Supplementary Fig. 4) and subsequently concentrated to the desired concentration.

*Ypt7_1-182 Q35C_–aGDP*. The protein (in 20 mM Hepes pH 7.5, 50 mM NaCl, 1 mM TCEP and 1 mM EDTA) was crystallized at a concentration of 12 mg ml^−1^ (hanging drop, 500 μl reservoir solution 0.5 M NH_4_NO_3_, 16% (w/v) PEG3350, 20 °C, 1 μl protein solution mixed with 1 μl reservoir solution). Multiple crystals were separated by transferring them into a drop of reservoir solution including 5 mM MgCl_2_ and subsequently flash cooled in liquid nitrogen.

*Ypt7_1-182 Q35C K38I Q68L_–aGTP*. The protein (in 20 mM Hepes pH 7.5, 50 mM NaCl, 5 mM MgCl_2_ and 1 mM TCEP) was crystallized at a concentration of 29 mg ml^−1^ (hanging drop, 500 μl reservoir solution 20% (w/v) PEG3350, 0.2 M LiCl, 10 mM yttrium(III) chloride, 4 °C, 1 μl protein solution mixed with 1 μl reservoir solution). Crystals grew readily within 12 h and were directly flash cooled in liquid nitrogen.

*Ypt7_1-182 Q35C_–aGppNHp*. The protein (in 20 mM Hepes pH 7.5, 100 mM NaCl, 2 mM DTE and 5 mM MgCl_2_) was crystallized at a concentration of 19 mg ml^−1^ (hanging drop, 750 μl reservoir solution 0.1 M Tris pH 7, 25% PEG 6000, 1 M LiCl, 20 °C, 1 μl protein solution mixed with 1 μl reservoir solution). For data collection, crystals were directly flash cooled in liquid nitrogen.

*Data collection and structure determination*. All diffraction data was collected from single crystals at beamline X10SA at the Swiss Light Source (Paul Scherrer Institute, Villigen, Switzerland). Indexing and data reduction was performed with XDS^42^. All structures were solved by molecular replacement with Phaser^43^ using the previously published structures of Ypt7:GDP (pdb 1ky3) and Ypt7:GppNHp (pdb 1ky2)^29^. Final cycles of model building and refinement were performed using COOT^44^ and REFMAC5 (ref. 45), respectively. Restraints dictionaries for modified nucleotides were obtained from PRODRG^46^ or directly from REFMAC5 (ref. 47) after manual addition of the link description in the pdb-file. Data statistics for data collection and refinement are summarized in Supplementary Table 3. All images of X-ray crystal structures were rendered with PyMol^48^.

### Cell culture and transfection

HeLa cells were maintained at 37 °C and 5% CO_2_ in high glucose minimum essential medium (MEM; 21969-035, Invitrogen) supplemented with 10% fetal bovine serum. For confocal microscopy, 2.0 × 10^5^ cells were cultured on 35 mm glass bottom dishes (MatTek, Ashland, MA) for 20 h before transfection. Transient plasmid expression was achieved by overnight transfection with X-treme GENE HP DNA transfection reagent (06366244001, Roche).

### Confocal fluorescence microscopy

Imaging of cells was performed in DMEM without phenol red (31053-028, Life Technology) by using an inverted confocal microscope Leica TCS SP2 or SP5 (for eGFP-Rab5_E47C_) equipped with a 63 × /1.4 HCX Plan Apo oil immersion lens and a temperature-controlled hood at 37 °C with 5% CO_2_.

### Microinjection of Rab5 proteins

For each experiment, about 50 HeLa cells were injected with the purified Rab5 proteins at concentrations of ∼10 mg ml^−1^ in prenylation buffer (50 mM Hepes, pH 7.2, 50 mM NaCl, 5 mM DTE and 2 mM MgCl_2_). The microinjection was performed with an Eppendorf Transinjector 5246 and Eppendorf micromanipulators 5171. In order to exclude mislocalization of injected Rab proteins due to missing or incomplete prenylation, GFP–Rab5_E47C_–aGDP, GFP–Rab5_E47C, Q79L_–aGTP and GFP–Rab5_E47C_–aGppNHp were prenylated *in vitro* before microinjection as described previously^37^.

## Additional information

**Accession codes:** Atomic coordinates have been deposited in the pdb (accession codes 4PHF (Ypt7–aGDP), 4PHG (Ypt7–aGTP) and 4PHH (Ypt7–aGppNHp)).

**How to cite this article:** Wiegandt, D. *et al*. Locking GTPases covalently in their functional states. *Nat. Commun.* 6:7773 doi: 10.1038/ncomms8773 (2015).

## Supplementary Material

Supplementary Information Supplementary Tables 1-14, Supplementary Tables 1-2, Supplementary Methods and Supplementary References

## Figures and Tables

**Table 1:** X-ray data statistics.

	**Ypt7**_**Q35C**_**–aGDP**	**Ypt7**_**Q35C, K38I, Q68L**_**–aGTP**	**Ypt7**_**Q35C**_**–aGppNHp**
Data collection*			
Space group	P2_1_2_1_2_1_ (19)	P2_1_ (4)	P2_1_ (4)
Cell dimensions			
a, b, c (Å)	49.31, 54.90, 60.47	32.97, 53.16, 46.91	60.57, 72.23, 82.34
α, β, γ (°)	90.0, 90.0, 90.0	90.0, 106.353, 90.0	90.0, 91.485, 90.0
Wavelength (Å)	1.0	1.0	0.92045
Resolution (Å)	40.65–1.95	45.01–1.9	48.18–2.35
Highest shell	2.05–1.95	2.0–1.9	2.45–2.35
*R*_sym_ (%)	14.1 (54.3)	7.0 (36.2)	5.8 (55.5)
*R*_meas_ (%)	14.8 (57.0)	8.3 (43.3)	6.2 (59.6)
I/*σ*(I)	12.41 (5.17)	11.15 (2.85)	18.28 (3.18)
Completeness (%)	100 (100)	99.5 (99.8)	99.9 (99.8)
Redundancy	12.1 (11.1)	3.3 (3.2)	6.9 (7.0)
			
Refinement			
Resolution	40.65–1.95	45.01–1.9	48.18–2.35
Highest shell	2.0–1.95	1.95–1.9	2.41–2.35
No. of reflections	11,832	11,694	28,232
*R*_work_ (%)	19.6 (22.9)	17.5 (23.3)	19.9 (29.2)
*R*_free_ (%)	24.7 (32.9)	23.2 (34.3)	26.3 (33.1)
No. of atoms			
Protein	1,266	1,361	5,155
Ligands	41	44	168
Water	60	52	17
B-factors			
Protein	25.8	31.4	75.6
Ligands	28.7	26.7	71.5
Water	26.7	29.2	58.1
R.m.s deviations			
Bond length (Å)	0.019	0.019	0.015
Bond angles (°)	1.859	1.910	1.672
PDB entry code	4PHF	4PHG	4PHH

PDB, Protein data bank; R.m.s., root mean squared.

^*^The datasets for Ypt7_Q35C_–aGDP, Ypt7_Q35C_–aGppNHp and Ypt7_Q35C, K38I, Q68L_–aGTP were each collected from a single crystal at beamline X10SA (Swiss Light Source, Villigen, Switzerland).
