# Supplementary material for: Locking GTPases covalently in their functional states
Source: Nat Commun. 2015 Jul 16;6:7773. doi: 10.1038/ncomms8773 (PMC4518245; doi:10.1038/ncomms8773)
Supplement: Supplementary Information — Supplementary Tables 1-14, Supplementary Tables 1-2, Supplementary Methods and Supplementary References [file ncomms8773-s1.pdf]

1. Supplementary Figures

1.1. Synthesis of the acryl-nucleotides

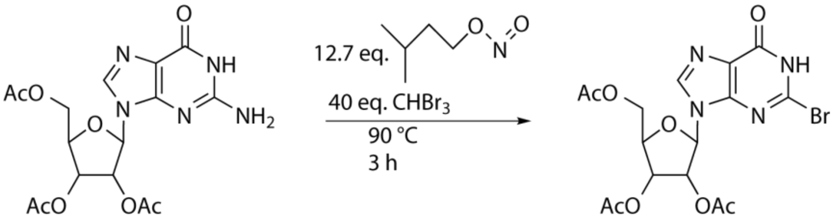

Supplementary Fig. 1-1: Activation of the purine C2-position

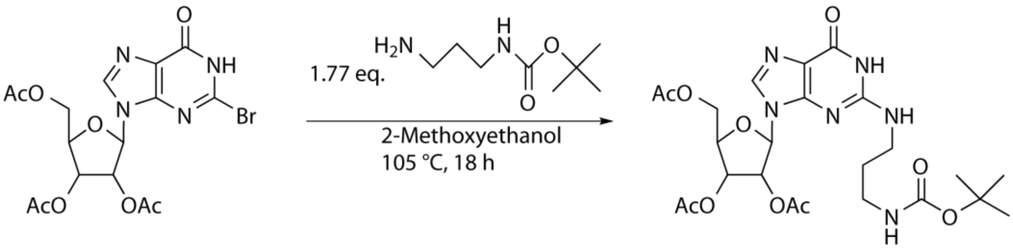

Supplementary Fig. 1-2: Introduction of the aminopropyl linker at the purine C2 position

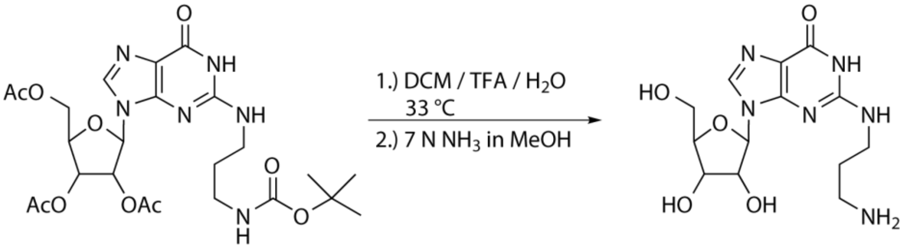

Supplementary Fig. 1-3: Removal of protecting groups

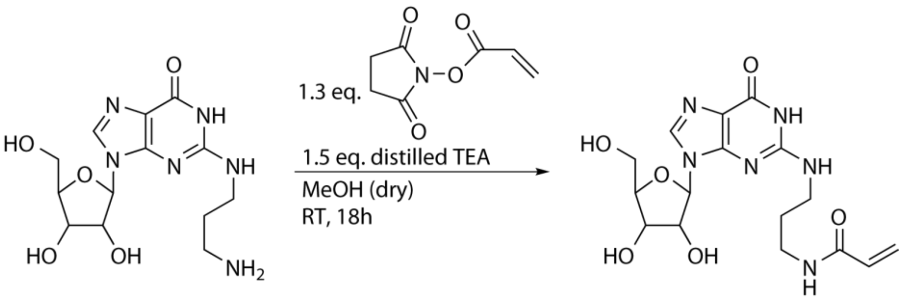

Supplementary Fig. 1-4: Introduction of the acryloyl group

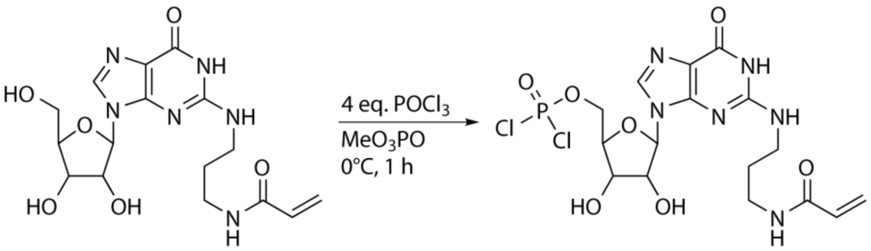

Supplementary Fig. 1-5: Phosphorylation of the 5’-hydroxyl group

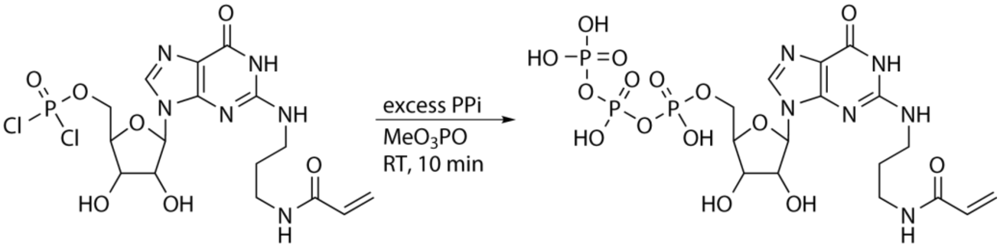

Supplementary Fig. 1-6: Phosphorylation to the triphosphate

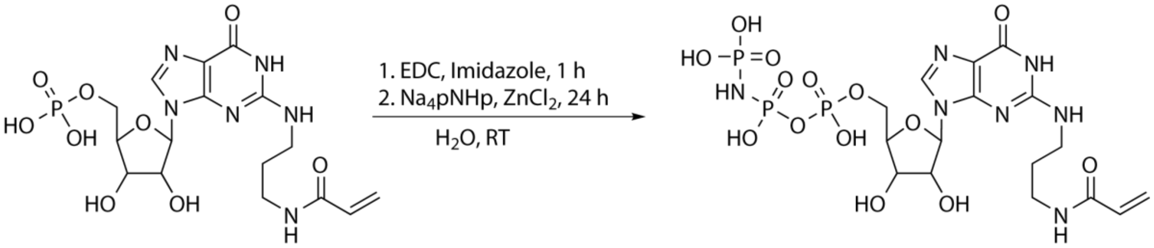

Supplementary Fig. 1-7: Preparation of 5’-(β,γ-imido)-triphosphate

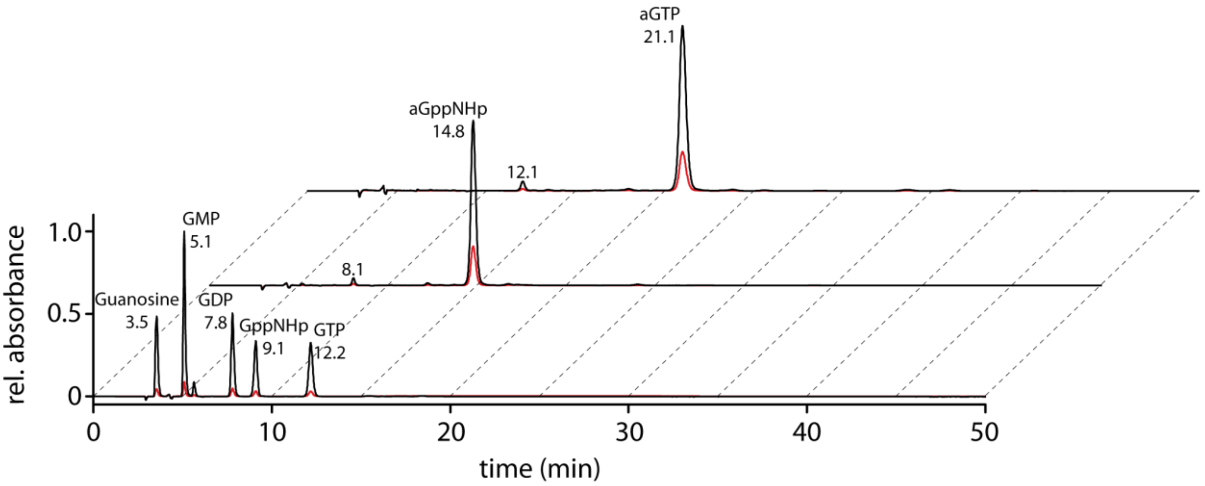

Supplementary Fig. 1-8: HPLC analysis of final compounds. The purity of the final compounds was analyzed via HPLC anaylsis (buffer: 50 mM KP_i pH 6.6, 10 mM tetra-n-butylammonium bromide, 16% acetonitrile, column: Prontosil 120-5-C18 AQ 5.0 µm 250x4.6mm, flow rate 1 ml/min). A standard mixture containing guanosine, guanosine-5’-monophosphate (GMP), guanosine-5’-diphosphate (GDP), guanosine-5’-triphosphate (GTP) and guanosine-5’-[β,γ-imido]-triphosphate (GppNHp) is shown in the lower trace and compared with aGTP (upper trace) and aGppNHp (middle trace; black - absorption at 254 nm, red - absorption at 295 nm). Retention times (in minutes) corresponding to each peak are indicated.

1.2. Mutation sites in Rab1b

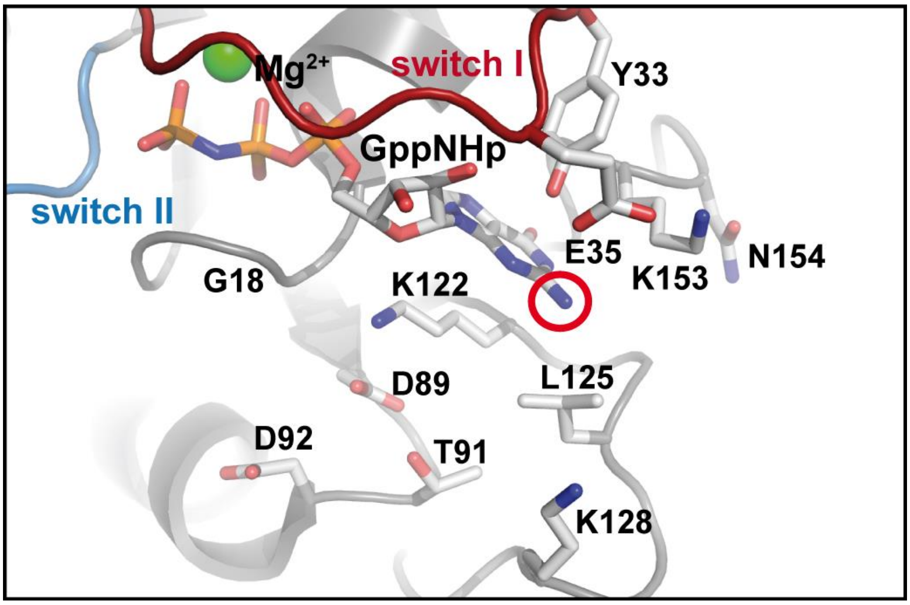

Supplementary Fig. 2: Mutation sites in Rab1b. All indicated amino acids in Rab1b were mutated to cysteine residues and tested in this publication for covalent modification with acrylGTP (GppNHp: Guanosine 5′-[β,γ-imido]triphosphate, switch I – red, switch II – blue, pdb 3nkv [1]). The modified N2 amino position for attachment of the acryl-linker is indicated by a red circle.

1.3. Mass spectra of small GTPases modified with aGTP

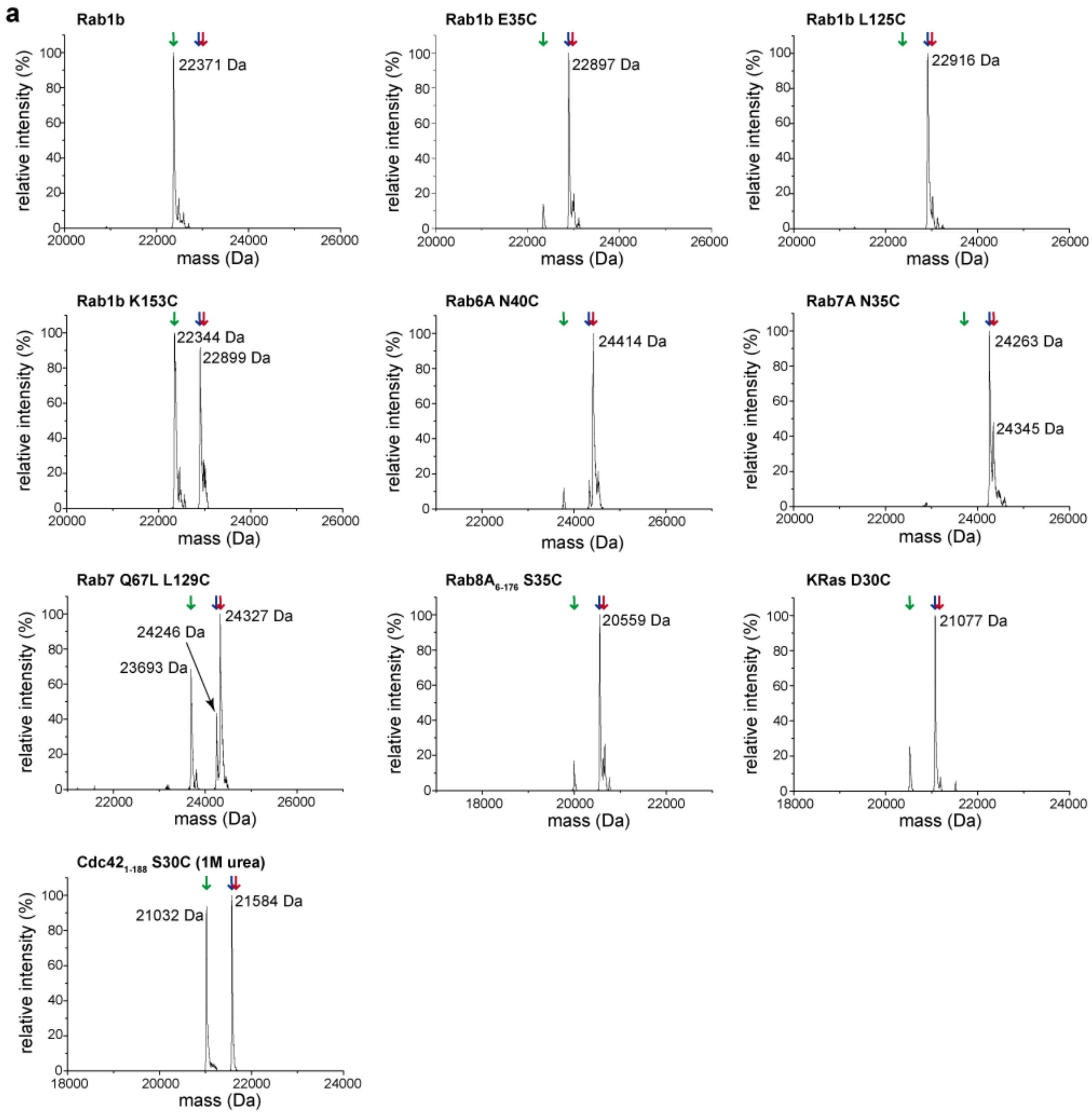

Supplementary Fig. 3: Mass spectra of modified small GTPases. (a) Mass spectra were measured after incubation of the respective small GTPase with acrylGTP as described in online methods. The arrows indicate unmodified small GTPases (green), aGDP-modified small GTPases (blue) and aGTP-modified GTPases (red). The calculated masses for the different proteins are: Rab1b 22365.3 Da; Rab1b E35C 22339.3 Da; Rab1b L125C 22355.3 Da; Rab1b K153C 22340.3 Da; Rab6A N40C 23776.0 Da; Rab7 N35C 23703.0 Da; Rab7 Q67L L129C 23688.9 Da; Rab8A$_{6\text{-}176}$ S35C 19998.0 Da; KRas$_{1\text{-}180}$ D30C 20521.4 Da; Cdc42$_{1\text{-}188}$ S30C 21020.2 Da. The covalent addition of aGuanosine, aGDP and aGTP induce a mass increase of 397.2 Da, 555.2 Da and 635.2 Da, respectively.

1.4. Mass spectra of $Ypt7_{1-182}$ mutants used for structure determination

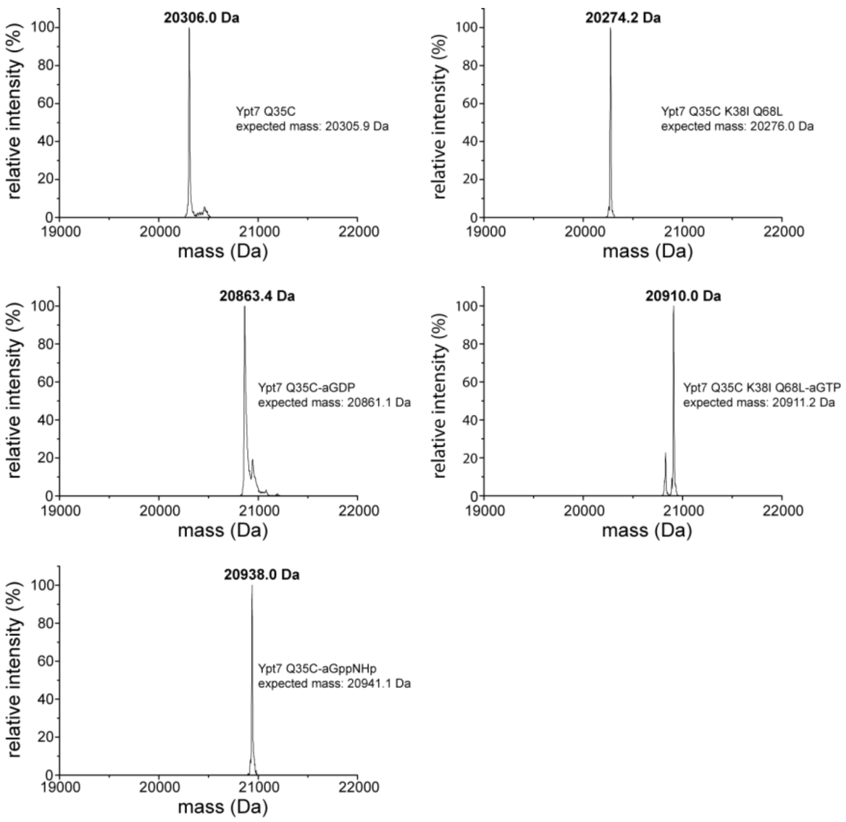

Supplementary Fig. 4: Mass spectra of $Ypt7_{1-182}$ mutants before and after covalent modification with aGDP, aGppNHp and aGTP. Observed masses for the different proteins are depicted above the corresponding peaks in the mass spectra, expected (calculated) masses are shown on the right side of each spectrum. The modified proteins were used for crystallization and structure determination.

1.5. Complex formation of DrrA and Rab1

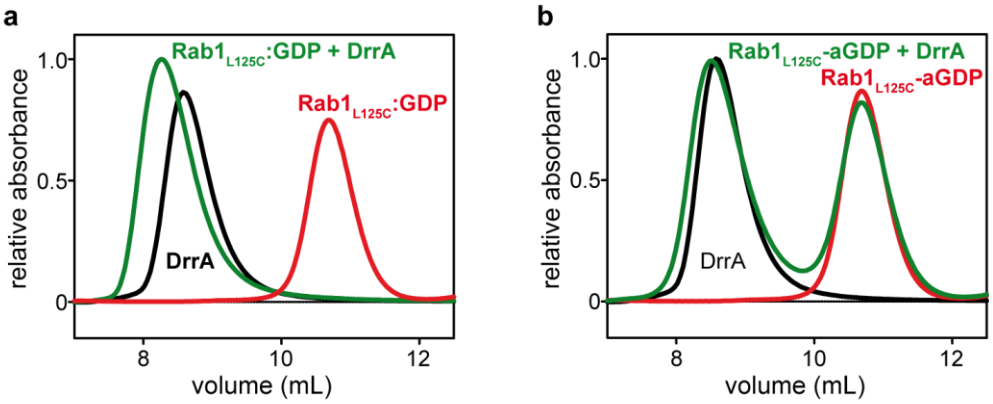

Supplementary Fig. 5: Complex formation of DrrA and Rab1 is only possible with non-covalently nucleotide bound Rab1. Guanine nucleotide exchange factors (GEFs) bind their target protein substrates preferentially in the nucleotide-free state. Therefore, a stable complex can only be formed starting with Rab1 non-covalently bound to GDP (**a**), but not with the covalently aGDP-locked form (**b**) as shown in these analytical gel filtration experiments. The experiments were performed in nucleotide-free buffer.

1.6. Generation of Rab1b-aGuanosine

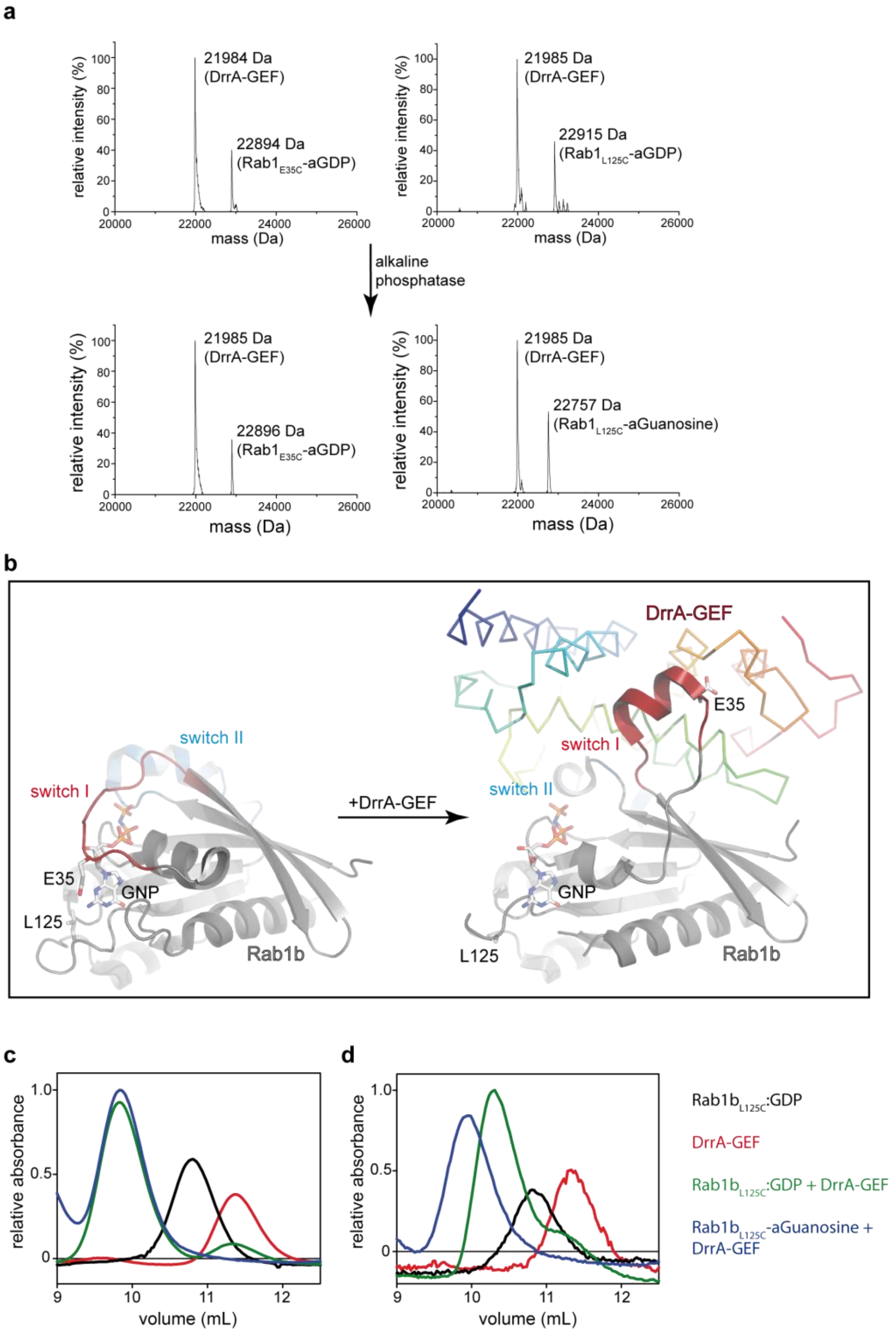

Supplementary Fig. 6: Generation of Rab1 in a quasi-nucleotide-free state. (**a**) Whereas incubation of stoichiometric amounts (each 1.25nmol) of Rab1b-aGDP and DrrA-GEF with 10 u alkaline phosphatase led to generation of Rab1b-aGuanosine in the case of $Rab1b_{L125C}$, this was not the case for $Rab1b_{E35C}$ (upper spectra before and lower spectra 20 hours after addition of alkaline phosphatase). (**b**) This difference can likely be attributed to the huge movement of the switch I region of Rab1 (grey cartoon, switch I and switch II are colored in red and blue, respectively) upon complex formation with DrrA-GEF (rainbow-colored ribbon), which is sterically hindered due to the covalent linkage in $Rab1b_{E35C}$-aGDP, but not $Rab1b_{L125C}$-aGDP. (**c**) In nucleotide-free buffer (20 mM Hepes pH 7.5, 50 mM NaCl, 1 mM TCEP, 1 mM $MgCl_2$), $Rab1b_{L125C}$ non-covalently bound to GDP and covalently bound to aGuanosine form stable complexes with DrrA-GEF in analytical gel filtration, whereas (**d**) addition of 100 µM GDP to the running buffer leads to a partial disruption of complex formation in case of $Rab1b_{L125C}$:GDP, but not $Rab1b_{L125C}$-aGuanosine. These results indicate that aGuanosine-bound Rab1 is a locked analogue of the nucleotide-free state of Rab1 (elution profiles were recorded at 280 nm in (**c**) and at 295 nm in (**d**) due to strong absorption of the buffer supplemented with GDP).

1.7. Interaction of Rab1 with GTPase activating proteins

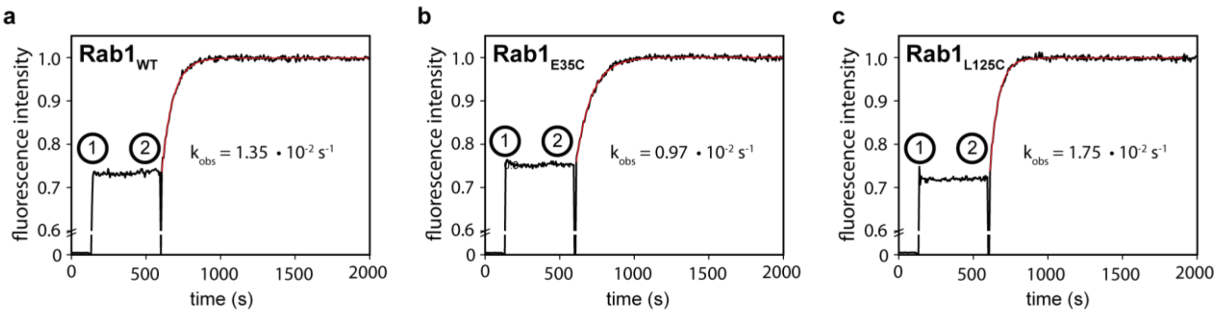

Supplementary Fig. 7: The interaction of TBC1D20 is not significantly affected by the Cys-residues introduced into Rab1b. 4 µM $Rab1b_{WT}$:GTP (a), $Rab1b_{E35C}$:GTP (b) and $Rab1b_{L125C}$:GTP (c) added to buffer (20 mM Hepes pH 7.5, 50 mM NaCl, 2 mM DTE, 2 mM $MgCl_2$) in step 1 were supplemented with 0.04 µM $TBC1D20_{1\text{-}362}$ (step 2) and GTP hydrolysis was monitored by the change in tryptophan fluorescence. The observed rate constants (k_{obs}) derived from single exponential fits (red lines) indicate no significant effects of the mutations in Rab1b on the interaction with TBC1D20.

1.8. *In vitro* Prenylation of Rab1b

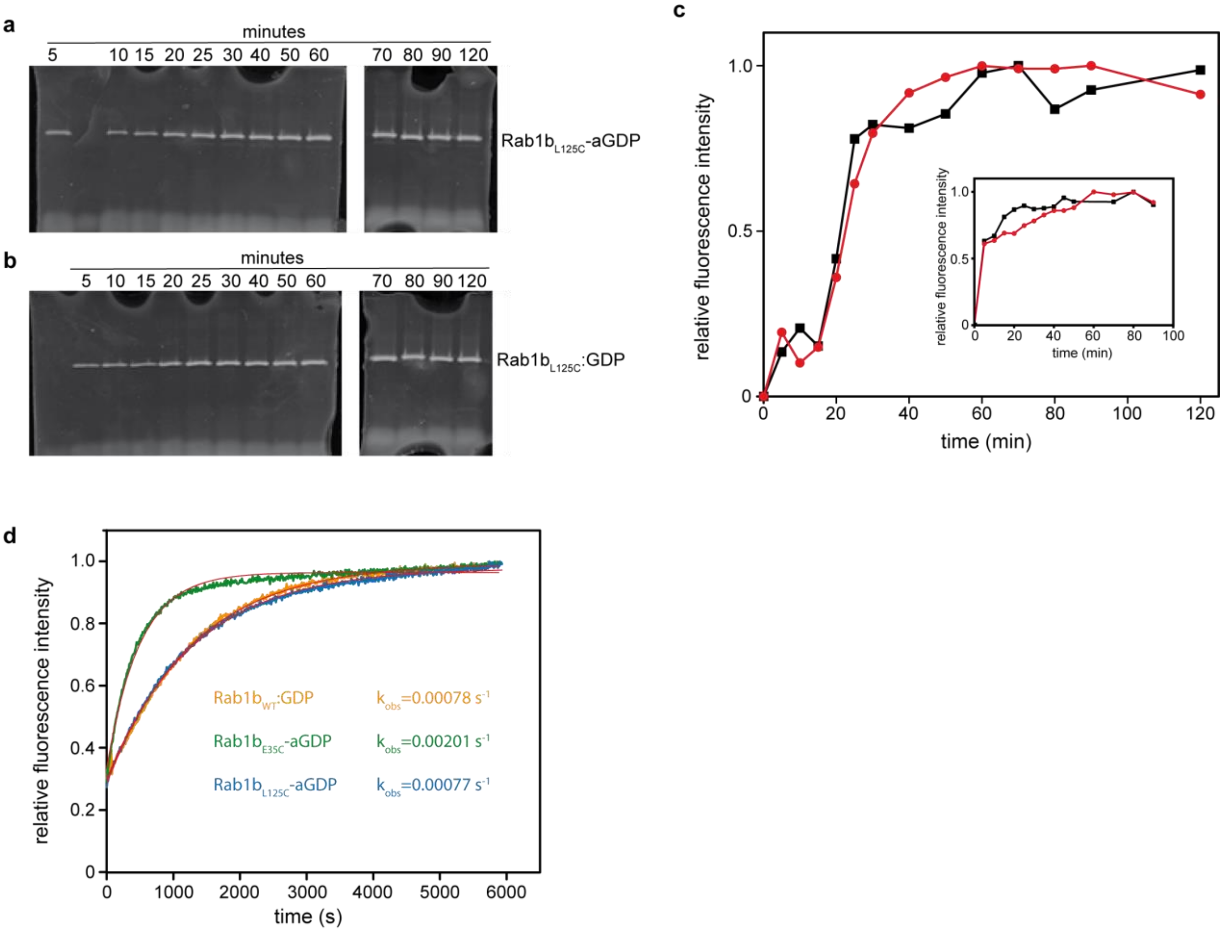

Supplementary Fig. 8: Prenylation of Rab1b. The prenylation of Rab1b$_{L125C}$-aGDP (**a**) and Rab1b$_{L125C}$:GDP (**b**) by REP1 and GGTase was followed via in gel fluorescence at different time points of the reaction. (**c**) Plot of the quantified in gel fluorescence against the reaction time for Rab1b$_{L125C}$-aGDP (red) and Rab1b$_{L125C}$:GDP (black) (inlet: Rab1b$_{E35C}$-aGDP (red) and Rab1b$_{E35C}$:GDP (black)). (**d**) The prenylation of 1µM Rab:REP complex by 400nM RabGGTase was quantified for Rab1b$_{WT}$:GDP, Rab1b$_{E35C}$-aGDP and Rab1b$_{L125C}$-aGDP using the change of fluorescence of NBD-FPP as described previously [2]. In this assay, Rab1b$_{E35C}$ is prenylated with a slightly faster rate (2.6x) compared to Rab1b$_{WT}$ and Rab1b$_{L125C}$.

1.9. Interaction of Rab1b with GDI

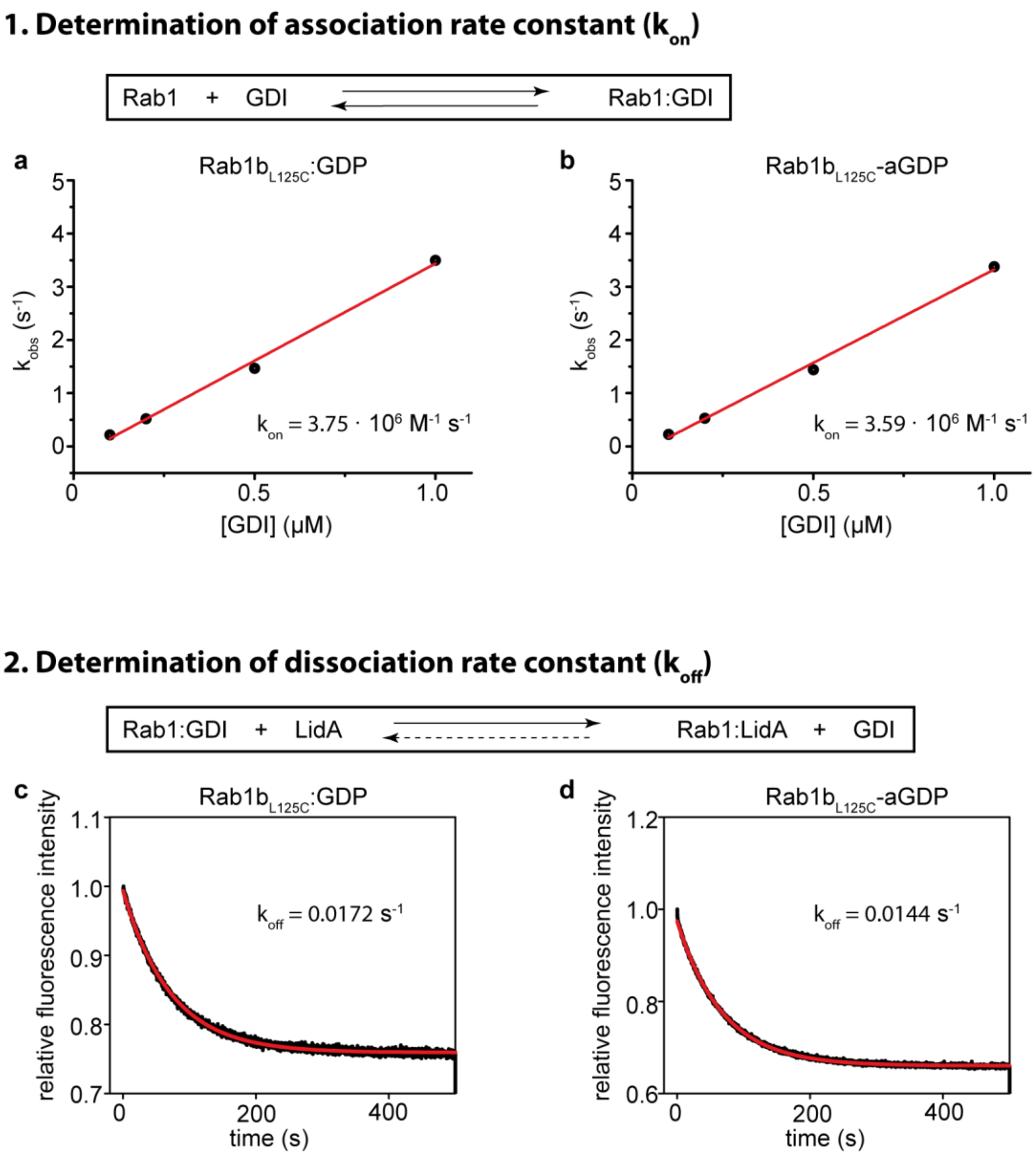

Supplementary Fig. 9: Interaction of Rab1b with GDI.
1. For determination of the association rate constants (k_{on}) of Rab1b and GDI, fluorescently labeled and farnesylated Rab1b was used as described previously[3]. The observed rate constants (k_{obs}) of the interaction between Rab1b$_{L125C}$:GDP (**a**) or Rab1b$_{L125C}$-aGDP (**b**) and GDI were determined using different concentrations of GDI. The slope of the linear fit (red) yielded k_{on}.
2. For determination of the dissociation rate constant, a displacement experiment of the Rab1b:GDI complex with excess of the effector protein LidA[3,4] was performed making use of the extraordinary high affinity of LidA:Rab1 complex[4]. Displacement of Rab1b$_{L125C}$:GDP:GDI (**c**) or Rab1b$_{L125C}$-aGDP:GDI (**d**) complexes by LidA was fitted using a single exponential equation to yield the dissociation rate constant k_{off} . The dissociation constant (K_D) was calculated from k_{off}/k_{on}.

1.10. Interaction of Rab1b$_{E35C}$ and Rab1b$_{L125C}$ with the effector Mical

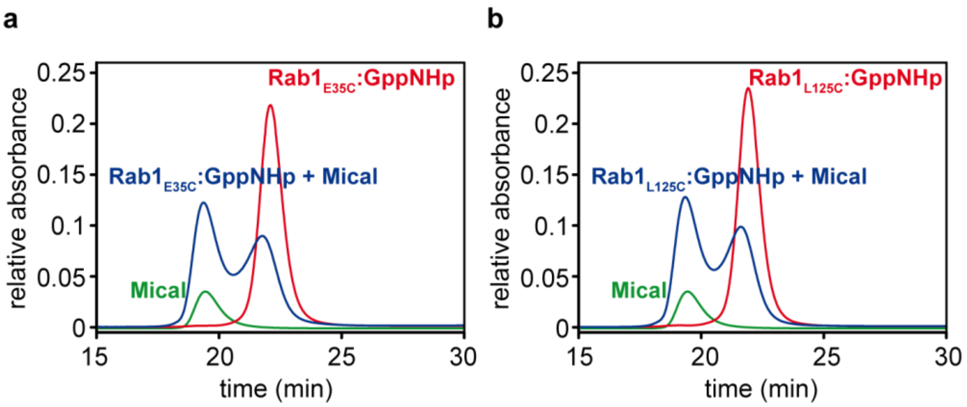

Supplementary Fig. 10: Interaction of Rab1b$_{E35C}$ and Rab1b$_{L125C}$ with the effector protein Mical. (**a**) Rab1b$_{E35C}$ and (**b**) Rab1b$_{L125C}$ bound non-covalently to GppNHp form complexes with the effector protein Mical as shown via analytical gel filtration (running buffer: 20 mM Hepes pH 7.5, 50 mM NaCl, 2 mM DTE, 2 mM $MgCl_2$). This indicates that the mutant Rab1 proteins behave similarly to wildtype Rab1 (see Figure 4 in the main manuscript).

1.11. Structural comparison of Ypt7 variants

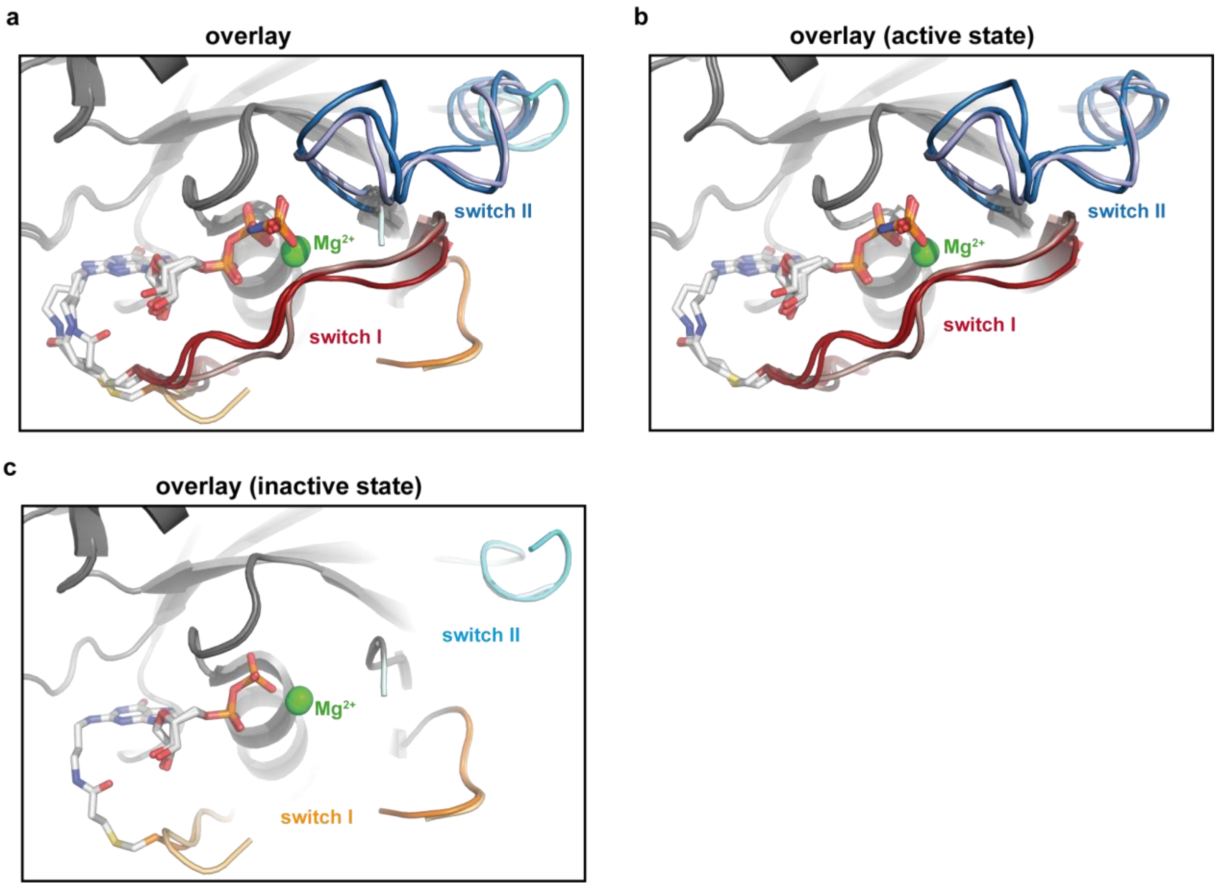

Supplementary Fig. 11: The conformation of the nucleotide binding pocket is essentially undisturbed by the covalent modification. The superposition of Ypt7 bound non-covalently to GDP (pdb 1KY3) and GppNHp (pdb 1KY3) with Ypt7-aGDP, Ypt7-aGTP and Ypt7-aGppNHp show that the covalently locked variants can adopt similar conformations of the nucleotide binding pocket as the native variants. (**a**) shows the superposition of all structures, (**b**) shows the superposition of Ypt7 in the active state conformation (*i.e.* Ypt7:GppNHp, Ypt7-aGTP and Ypt7-aGppNHp) and (**c**) shows the superposition of the inactive (*i.e.* Ypt7:GDP and Ypt7-aGDP) states (switch I is colored in red (Ypt7-aGTP and Ypt7-aGppNHp), light red (Ypt7:GppNHp), orange (Ypt7-aGDP) or light orange (Ypt7:GDP), switch II is colored in blue (Ypt7-aGTP and Ypt7-aGppNHp), violet (Ypt7:GppNHp), cyan (Ypt7-aGDP) or light cyan (Ypt7:GDP).

1.12. F_o-F_c omit maps

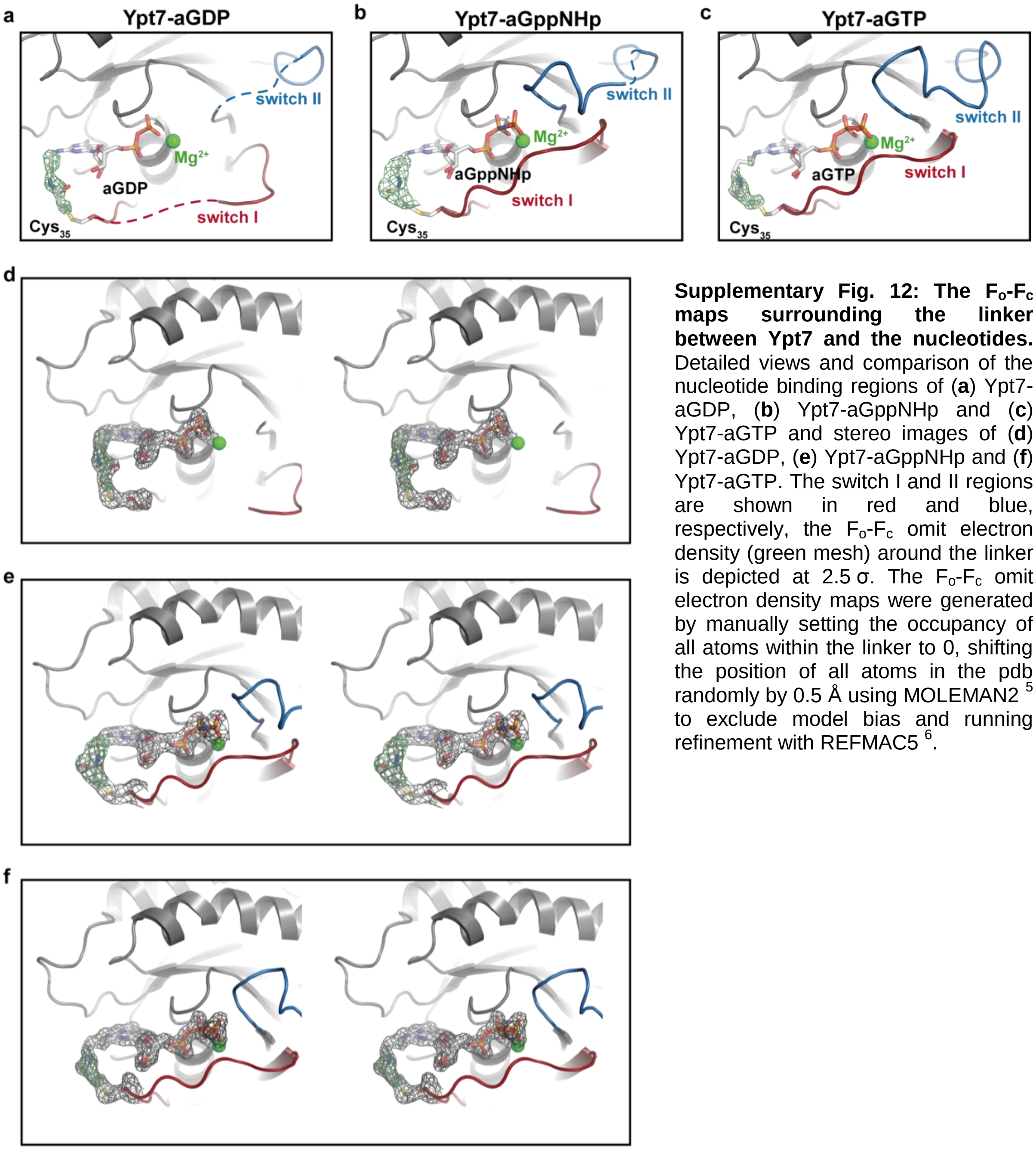

Supplementary Fig. 12: The F_o-F_c maps surrounding the linker between Ypt7 and the nucleotides. Detailed views and comparison of the nucleotide binding regions of (**a**) Ypt7-aGDP, (**b**) Ypt7-aGppNHp and (**c**) Ypt7-aGTP and stereo images of (**d**) Ypt7-aGDP, (**e**) Ypt7-aGppNHp and (**f**) Ypt7-aGTP. The switch I and II regions are shown in red and blue, respectively, the F_o-F_c omit electron density (green mesh) around the linker is depicted at 2.5 σ. The F_o-F_c omit electron density maps were generated by manually setting the occupancy of all atoms within the linker to 0, shifting the position of all atoms in the pdb randomly by 0.5 Å using MOLEMAN2 [5] to exclude model bias and running refinement with REFMAC5 [6].

1.13. Model of GEF-mediated intracellular targeting of Rab proteins

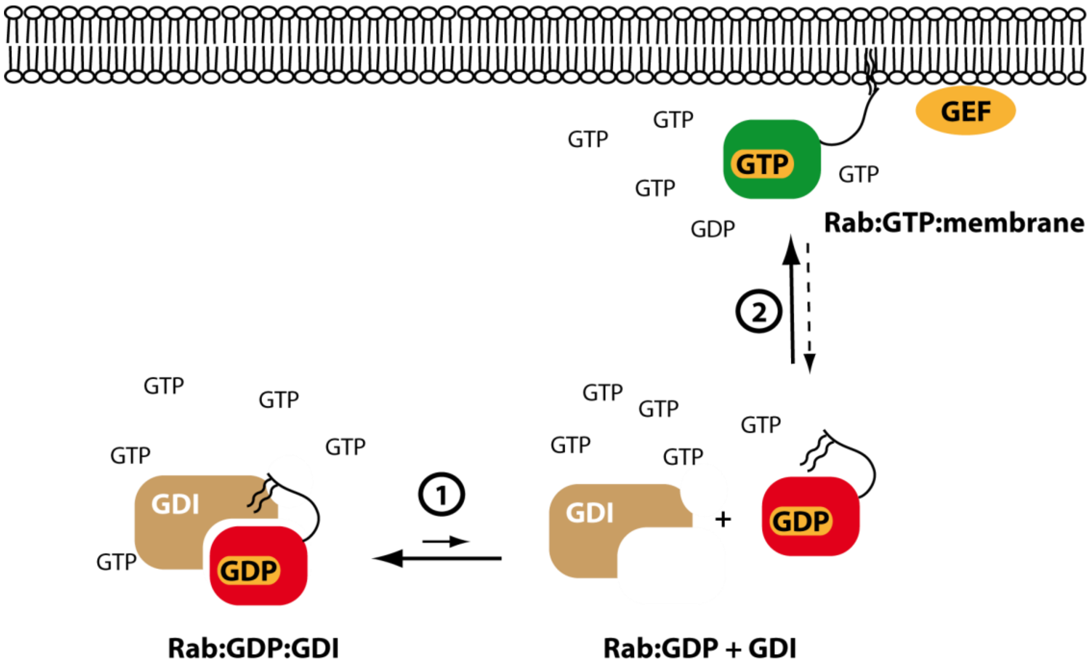

Supplementary Fig. 13: Model of GEF mediated intracellular targeting of Rab proteins. Rab proteins in the inactive GDP-bound form exist in the cytosol mainly in the GDI bound state. However, a small fraction is dissociated from GDI as indicated in equilibrium 1. GEF proteins localized at a certain membrane in the cell can pull out free Rab proteins from this equilibrium making use of the high relative concentration of GTP compared to GDP in the cell[7] and the low affinity of GDI towards GTP-bound Rab proteins (equilibrium 2)[3,8]. This model suggests that GEF proteins olay a decsive role in the localization of a Rab proteins to a specific membrane in the cell.

1.14. Prenylation of Rab5

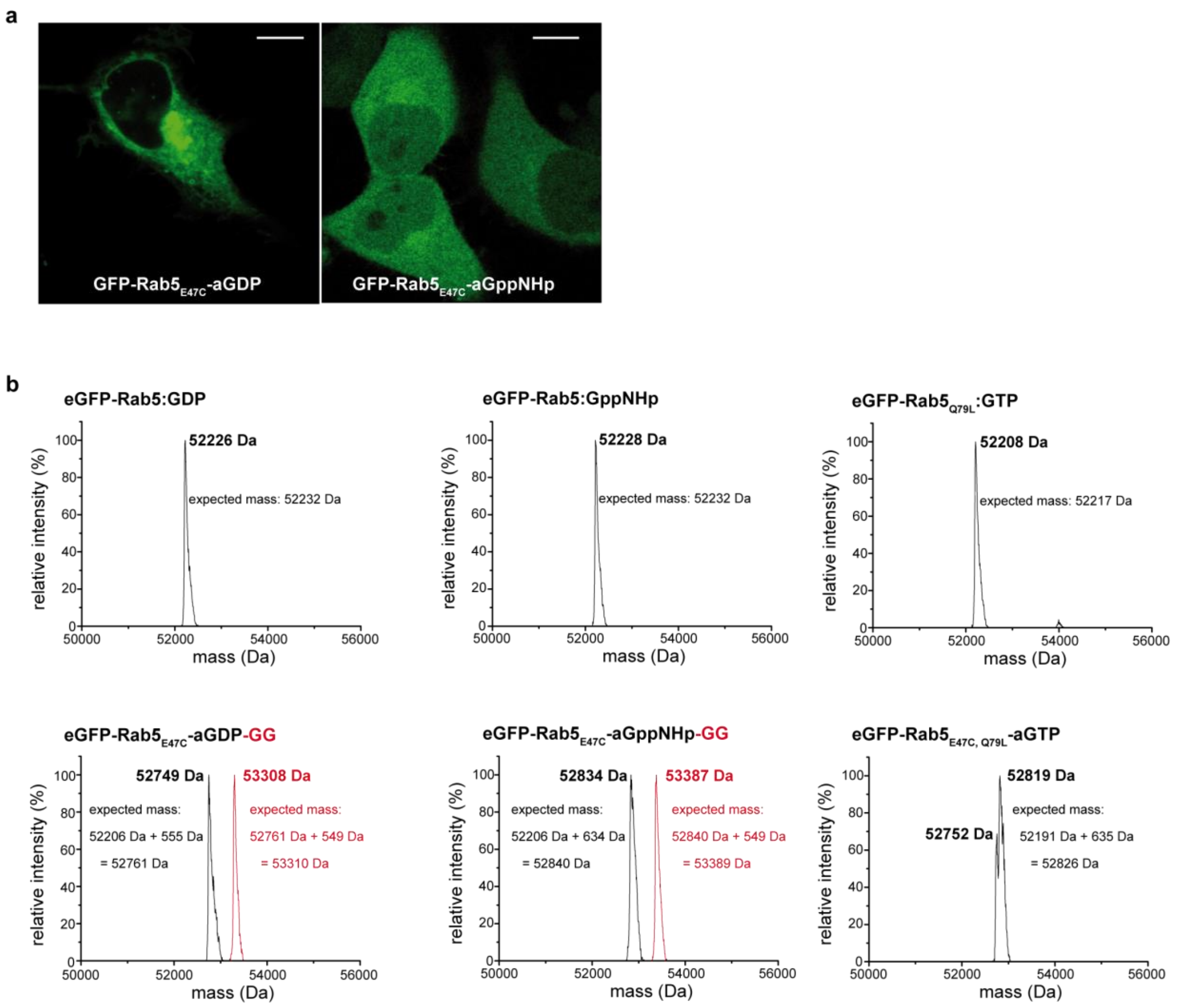

Supplementary Fig. 14: Intracellular localization of different Rab5 constructs. (**a**) GFP-Rab5 covalently bound to aGDP (left) or aGppNHp (right) were microinjected into cells without prior prenylation *in vitro*. Rab5-aGDP shows localization to the Golgi apparatus similarly to Rab5-aGDP prenylated *in vitro* before microinjection (see Figure 6 in the main article for comparison). Rab5-aGppNHp in contrast does not localize to any membranous structures inside the cell but is distributed throughout the cell including the nucleus. This indicates that prenylation does not occur inside the cell in case of the locked aGppNHp-bound Rab protein (scale bar 10 µm). (**b**) Mass spectra of all eGFP-Rab5 constructs used for the microinjection experiments. The lower panel shows the mass spectra of acryl-nucleotide modified proteins (black) and the geranylgeranylated proteins (red). The covalent attachment of aGDP (555.2 Da), aGppNHp (634.2 Da), aGTP (635.2 Da) and two geranylgeranyl (GG) moieties (549 Da) can be clearly seen in the mass spectra.

2. Supplementary Tables

2.1. Proteins used in this study

Supplementary Table1: Proteins used in this study listing expression plasmids and final storage buffer.

Protein	Plasmid (source)	Final gel filtration buffer
Rab1b	pMAL Rab1b[9]	20 mM Hepes pH 7.5, 50 mM NaCl, 1 mM TCEP, 1 mM $MgCl_2$ 10 µM GDP
Rab1b G18C	pMAL Rab1b G18C (*)	as above
Rab1b Y33C	pMAL Rab1b Y33C (*)	as above
Rab1b E35C	pMAL Rab1b E35C (*)	as above
Rab1b E35C Q67L	pMAL Rab1b E35C Q67L (*)	as above
Rab1b E35C Q67A	pMAL Rab1b E35C Q67A (*)	as above
Rab1b D89C	pMAL Rab1b D89C (*)	as above
Rab1b T91C	pMAL Rab1b T91C (*)	as above
Rab1b D92C	pMAL Rab1b D92C (*)	as above
Rab1b K122C	pMAL Rab1b K122C (*)	as above
Rab1b L125C	pMAL Rab1b L125C (*)	as above
Rab1b L125C CVIL	pMAL Rab1b L125C CVIL (*)	as above
Rab1b K128C	pMAL Rab1b K128C (*)	as above
Rab1b K153C	pMAL Rab1b K153C (*)	as above
Rab1b N154C	pMAL Rab1b N154C (*)	as above
Rab6A N40C	pET19mod Rab6A N40C (*)	20 mM Hepes pH 7.5, 200 mM NaCl, 1 mM TCEP, 1 mM $MgCl_2$, 10 µM GDP
Rab7A N35C	pMAL Rab7A N35C (*)	as above
Rab7A Q67L L129C	pMAL Rab7A L129C Q67L	as above
$Rab8A_{6\text{-}176}$ S35C	pET19mod $Rab8A_{6\text{-}176}$ S35C (*)	as above
$Ypt7_{1\text{-}182}$ Q35C	pET19mod $Ypt7_{1\text{-}182}$ Q35C (*)	as above
$Ypt7_{1\text{-}182}$ Q35C Q68L	pET19mod $Ypt7_{1\text{-}182}$ Q35C Q68L (*)	as above
$Ypt7_{1\text{-}182}$ Q35C K38I Q68L	pET19mod $Ypt7_{1\text{-}182}$ Q35C K38I Q68L (*)	as above
$Cdc42_{1\text{-}188}$ S30C	pOPINM $Cdc42_{1\text{-}188}$ S30C (*)	as above
GFP-$Rab5_{WT}$	pOPiN nGFP Rab5a WT (*)	20 mM Hepes pH 7.5, 200 mM NaCl, 1 mM TCEP, 2 mM $MgCl_2$
GFP-$Rab5_{E47C}$	pOPiN nGFP Rab5a E47C (*)	as above
GFP-$Rab5_{E47C,\ Q79L}$	pOPiN nGFP Rab5a E47C Q79L (*)	as above
GFP-$Rab5_{Q79L}$	pOPiN nGFP Rab5a Q79L (*)	as above
$KRas_{1\text{-}180}$ D30C	pTAC KRas D30C (*)	20 mM Hepes pH 7.2, 200 mM NaCl, 1 mM $MgCl_2$, 1 mM TCEP, 10 µM GDP
$DrrA_{340\text{-}533}$	pET19mod $DrrA_{340\text{-}533}$[9]	20 mM Hepes pH 8.0, 50 mM NaCl, 2 mM DTE
$LidA_{201\text{-}583}$	pOPINF $LidA_{201\text{-}583}$[4]	as above
$TBC1D20_{14\text{-}305}$	pOPINF $TBC1D20_{14\text{-}305}$[10]	20 mM Hepes pH 7.5, 50 mM NaCl, 1 mM DTE
$REP1_{1\text{-}650}$	pFBHTb $ratREP1_{1\text{-}650}$	25 mM Hepes pH 7.2, 40 mM NaCl, 5 mM DTE
RabGGTase I (α- and β-subunit)	pET30 RabGGTase α[11]<br>pGATEV RabGGTase β[11]	50 mM Hepes pH 7.2, 10 mM NaCl, 2 mM β-mercaptoethanol
GDI-I	pFAST BAC GDI I[9]	20 mM Hepes pH 7.5, 50 mM NaCl, 5 mM β-mercaptoethanol
Mical-$3_{1841\text{-}1990}$	pOPINF Mical-$3_{1841\text{-}1990}$[12]	20 mM Hepes pH 7.5, 100 mM NaCl, 2 mM DTE

(*) - this study

2.2. Percentage of modification of small GTPases using acrylGTP

Supplementary Table 2: Percentage of modification of small GTPases using acrylGTP estimated from mass spectra in Supplementary Fig. 3.

GTPase	incubation time (hours)	Completeness of reaction
Rab1 WT	23	0 %
Rab1 E35C	18	~ 90 %
Rab1 L125C	23	100 %
Rab1 K153C	23	~ 50 %
Rab5 E47C	20	100 %
Rab6 N40C	18	~ 90 %
Rab7 N35C	18	~ 100 %
Rab7 Q67L L129C	21	~ 70 %
Rab8 S35C	18	~ 80 %
Ypt7 Q35C	18	100 %
KRas D30C	25	~ 80 %
Cdc42 S30C(*)	18	~ 50 %

(*) - incubation was performed in the presence of 1 M urea to increase the rate of conversion, the completeness without urea was less than 10 % after 18 h.

3. Supplementary Methods

3.1. Synthesis of acryl-guanosine derivatives

Activation of the purine C2-position (Supplementary Fig. 1-1)

A modified form of the Sandmeyer reaction according to Cadogan et al.[13] was used for this step. 2´,3´,5´-Triacetyl guanosine (50 g or 122 mmol) was dissolved in a mixture of bromoform (420 ml) and isoamyl nitrite (206 ml) and heated to 90 °C for 2 h in the dark. At this time, RP-HPLC showed that the educt had reacted completely. After removal of isoamyl nitrite and bromoform under vacuum, the product was isolated by chromatography on 4.5 kg silica gel using an ethyl acetate-methanol mixture (19:1)-to give 35.8 g (62% yield) of the crude product as an orange-yellow powder.

^{1}H-NMR spectrum, HH-COSY spectrum

1H-NMR (400 MHz, D_2O/CD_3CN 1:1): δ [ppm], J [Hz]: 2.09 (s, 6H, 18-H, 19-H), 2.15 (s, 3H, 20-H), 4.28 (s, H_2O), 4.39 (ddd, J1: 3.90, J2: 12.38, J3:17.02, 2H, 5-H), 4.51 (dt, J13.19, J2, 4.78, J3, 4.82, 1H, 4-H), 5.65 (t, J1: 5.45, J2: 5.45, 1H, 3-H), 5.87 (dd, J1: 5.09, J2: 5.68, 1H, 2-H), 6.24 (d, J:4.98, 1H, 1-H), 8.15 (s, 1H, 6-H), 8.21 (s, 1H, 13-H)

^{13}C-NMR Spektrum

^{13}C-NMR (400 MHz, D_2O/CD_3CN 1:1): δ [ppm]: 22.59 (s, 20-C), 22.75 (s, 19-C), 22.99 (s, 18-C), 65.88 (s, 5-C), 73.16 (s, 3-C), 76.11 (s, 2-C), 82.96 (s, 4C), 89.36 (s, 1-C), 121.37 (s, CD_3CN), 127.30 (s, 10-C), 142.65 (s, 13-C), 148.89 (s, 9-C), 151.41 (s, 7-C), 160.72 (s, 11-C), 173.89 (s, 16-C), 174.18 (s, 18-C), 175.09 (s, 17-C)

High-resolution *electrospray* ionization mass spectral data (HR-ESI-MS): 473.03031 and 475.02823 Da (the two different masses result from the stable isotopes ^{79}Br und ^{81}Br (natural abundance 50.7 and 49.3 %, respectively). theoretical mass of the charged ions 473.03025 and 475.02821 Da, deviation: 0.12152 und 0.06031 ppm, respectively)

Introduction of the aminopropyl linker at the purine C2 position (Supplementary Fig. 1-2)

2´,3´,5´-Triacetyl-2 bromopurine nucleoside (4 g; 8.47 mmol) was dissolved in 2-methoxy ethanol (65 ml) and heated to 105 °C. Over a period of 2 h and at intervals of 10 min, *tert*-butyl-3-aminopropyl-carbamate (14.4 mmol) was added with stirring. After complete addition of the reagent the temperature was held at 105 °C for a further 4 h, after which time RP-HPLC indicated that no further reaction occurred. After cooling, removal of solvent *in vacuo*

led to a brown syrup, which was dissolved in 200 ml H_2O/acetonitrile and lyophilized to give the crude product as a brown powder. This was used without purification for the next step.

^{1}H-NMR spectrum, HH-COSY spectrum

^{1}H-NMR (400 MHz, D_2O/CD_3CN 1:1): δ [ppm], J [Hz]: 1.40 (s, 9H, 28-H, 29-H, 30-H), 1.77 (p, J1=J2: 6.56, J3=J4:6.46, 2H, 23-H), 2.03 (s, 1H, 19-H), 2.12 (s, 1H, 18-H), 2.14 (s, 1H, 20-H), 3.12 (t, J1=J2:6.73, 2H, 24-H), 3.43 (tq, J1=J2: 6.80, J3=J4=J5: 13.78, 2H, 22-H), 4.26 (s, H_2O), 4.27 (m, 4-H), 4.44 (ddd, J1: 3.47, J2: 10.62, J3: 15.24, 2H, 5-H), 5.75 (t, J1=J2: 6.43, 1H, 3-H), 6.00 (dd, J1: 4.00, J2: 5.64, 1H, 2-H), 6.05 (d, J:3.60, 1H 1-H), 7.81 (s, 1H, 13-H)

^{13}C-NMR Spektrum

^{13}C-NMR (400 MHz, D_2O/CD_3CN 1:1): δ [ppm]: 22.59 (s, 20-C), 22.66 (s, 19-C), 22.79 (s, 18-C), 30.44 (s, 3C, 28-C, 29-C, 30-C), 41.24 (s, 23-C), 58.14 (s, 22-C oder 24-C), 65.76 (s, 5-C), 72.75 (s, 3-C), 75.53 (s, 2-C), 81.85 (s, 4-C), 89.97 (s, 1-C), 121.31 (s, CD_3CN), 140.70 (s, 13-C)

HR-ESI-MS: 567.23065 Da (theoretical mass of the charged ion 567.24029 Da, deviation: 17 ppm)

Removal of protecting groups (Supplementary Fig. 1-3)

The crude product from the preceding step (4.9 g) was dissolved in dichloromethane (120 ml) containing 13.7 % (v/v) trifluoroacetic acid and 0.6 % (v/v) H_20. The solution was held at 33 °C for 7.5 h to remove the Boc protecting group. After removal of solvent *in vacuo*, the brown residue was dissolved in 7 M ammonia in methanol (60 ml) and held at RT for 6 h. Solvent and ammonia were removed *in vacuo*, after which purification was achieved on silica gel (120 g). After washing with acetonitrile/H_2O (9:1), the product was eluted using acetonitrile/H_2O (3:1). The product was obtained in a yield of 68 % relative to the bromo-purine starting material.

^{1}H-NMR spectrum, HH-COSY spectrum

^{1}H-NMR (400 MHz, D_2O/CD_3CN 1:1): δ [ppm], J [Hz]: 1,95 (qd, J1=J2: 6.70, J3: 6.58, J4: 13.24, 2H, 23-H), 3.04 (t, 2H, 24-H), 3.44 (t, 2H, 22-H), 3.76 (ddd, J1: 4.04, J2: 12.43, J3: 17.08, 2H, 5-H), 4.10 (td, J1=J2: 3.43, J3: 4.40, 1H, 4-H), 4.28 (s, H_2O), 4.34 (dd, J1: 4.58, J2: 5.23, 1H, 3-H), 4.69 (t, J1=J2: 5.34, 1H, 2-H), 5.84 (d, J:5.34, 1H, 1-H), 7.89 (s, 1H, 13-H)

^{13}C-NMR Spektrum

^{13}C-NMR (400 MHz, D_2O/CD_3CN 1:1): δ [ppm]: 29.45 (s, 23-C), 39.89 (s, 24-C), 40.47 (s, 22-C), 64.29 (s, 5-C), 73.19 (s, 3-C), 76.34 (s, 2-C), 87.65 (s, 4-C), 90.68 (s, 1-C), 119.22 (s, 10-C), 121.40 (s, CD_3CN), 140.36 (s, 13-C), 154.18 (s, 9-C), 155.66 (s, 7-C), 161.31 (s, 11-C)

HR-ESI-MS: 341.15679 Da (theoretical mass of the charged ion 341.15718 Da, deviation: 1,119 ppm).

Introduction of the acryloyl group (Supplementary Fig. 1-4)

2-Aminopropyl guanosine (1 g; 2.94 mmol) prepared in the previous step was stirred in dry methanol (clear solution not obtained), and this suspension was treated with dry and freshly distilled triethylamine (611 µl; 4.41 mmol). After addition of acrylic acid-N-hydroxysuccinimide ester (647 mg; 3.82 mmol) the suspension was stirred for 18 h at RT. After removal of solvent *in vacuo*, the residue was purified by chromatography on 300 ml of silica gel using acetonitrile/H_20 as eluent. 368.1 mg of the product was obtained (yield 31 %).

^{1}H-NMR spectrum, HH-COSY spectrum

^{1}H-NMR (400 MHz, D_2O/CD_3CN 1:1): δ [ppm], J [Hz]: 1.83 (p, J1=J2=J3=J4: 6.90, 2H, 23-H),3.32 (t, J1=J2: 6.82, 2H, 22-H), 3.39 (dt, J1: 2.09, J2: 6.84, J3: 6.67, 2H, 24-H), 3.77 (ddd, J1: 4.06, J2: 12.39, J3: 17.01, 2H, 5-H), 4.10 (dd, J1: 4.46, J2: 8.05, 1H, 4-H), 4.26 (s, H_2O), 4.36 (dd, J1: 4.41, J2: 5.43, 1H, 3-H), 4.73 (t, J1=J2: 5.29, 1H, 2-H), 5.69 (dd, J1: 1.80, J2: 9.98, 1H, 27-H), 5.85 (d, J: 5.30, 1H, 1-H), 6.20 (m, 2H, 28-H), 7.89 (s, 1H, 13-H)

^{13}C-NMR spectrum

^{13}C-NMR (400 MHz, D_2O/CD_3CN 1:1): δ [ppm]: 30.94 (s, 23-C), 39.56 (s, 24-C), 41.27 (s, 22-C), 64.34 (s, 5-C), 73.16 (s, 3-C), 76.15 (s, 2-C), 87.59 (s, 4-C), 91.00 (s, 1-C), 121.25 (s, CD_3CN), 129.43 (s, 27-C), 133.20 (s, 28-C), 140.69, (s, 13-C), 155.49 (s, 9-C), 161.34 (s, 7-C), 170.35 (s, 11-C), 183.89 (s, 26-C)

HR-ESI-MS: 395.16707 Da (theoretical mass of the charged ion 395.16736 Da, deviation: 0.732 ppm).

Phosphorylation of the 5'-hydroxyl group (Supplementary Fig. 1-5)

The N2-linked acryloyl guanosine derivative obtained in the previous step (316 mg) was suspended in dry trimethylphosphate (11 ml) under dry argon gas at 0 °C (method of Yoshikawa et al.[14]). After addition of $POCl_3$ (292 µl), the reaction vessel was removed from the ice bath and stirred for 1 h at RT. Excess $POCl_3$ was removed by evaporation *in vacuo*.

Reversed phase HPLC using tributylammonium bromide as a counterion in the running buffer indicated that the reaction was essentially complete after 1 h.
If the object of the phosphorylation was to generate the monophosphate (aGMP), the reaction mixture was treated with buffer and then purified by ion exchange chromatography on Q-Sepharose using a gradient of triethylammonium bicarbonate in water.

Resulting impurities with triethylammonium bicarbonate could not be completely removed from the product, therefore NMR-spectra for this and the subsequent steps cannot be provided. Instead, the compounds used in this study have been co-crystallized in complex with Ypt7 (see main article).

HR-ESI-MS: 475.13334 Da (theoretical mass of the charged ion 475.13369 Da, deviation: 0.737 ppm).

Phosphorylation to the triphosphate (Supplementary Fig. 1-6)
For the preparation of the triphosphate derivative of acryloyl guanosine, the phosphorodichloridate solution in trimethyl phosphate ensuing from the previously described reaction was treated after evaporation to remove excess $POCl_3$ with 10 equivalents of the tributylammonium salt of inorganic pyrophosphate dissolved in DMF[15]. This was prepared in the following manner: Disodium pyrophosphate (250 mg; 1.1 mmol) was dissolved in H_20 (20 ml). This solution was applied to a column of Dowex-50 in the pyridinium form (obtained by washing the H^+ form with 500 ml of 50 % aqueous pyridine followed by extensive washing). The column was eluted with MeOH/H_20 and the pyrophosphate-containing eluate was treated with tri-n-butylamine (5 ml), and evaporated on a rotary evaporator. The residue was dissolved in Pyridin/DMF (1:3) and evaporated to dryness (repeated twice). The residue was dissolved in 5 ml dry tri-n-butylamine and evaporated to dryness before dissolving in 5 ml of dry DMF.
After addition of the tributylammonium pyrophosphate to the crude phosphorodichloridate solution in trimethyl phosphate prepared as described above, the mixture was allowed to stand for 10 minutes and was then quenched by addition of 50 mM triethylammonium bicarbonate. Purification was achieved on a column of Q-Sepharose FF using a gradient of the same buffer. After pooling of fractions containing the product, solvent and buffer were removed by evaporation on a rotary evaporator. The oily residue was taken up in methanol, followed by removal of solvent and buffer by evaporation (repeated 3x).

HR-ESI-MS: 635.06621 Da (theoretical mass of the charged ion 635.06635 Da, deviation: 0.217 ppm).

Preparation of the 5'-[β,γ-imido]-triphosphate (Supplementary Fig. 1-7)

aGMP (25 mg, 53 µmol) was dissolved in an aqueous mixture of 950 µL BisTris (1.5 M, pH = 6.5) and 105 µL imidazole buffer (3 M, pH = 6.0). EDC hydrochloride (202 mg, 1.06 mmol) was added and the mixture was shaken. The formed clear solution was slightly agitated at room temperature for 1 h, after which HPLC analysis indicated > 90 % conversion to the imidazolate. Sodium imidodiphosphate (182 mg, 686 µmol), 1.06 mL imidazole buffer (3 M, pH = 6.0) and 115 µL aqueous zinc chloride solution (1 mg/mL, 844 µmol) were added. The reaction mixture was sonicated for 5 min followed by agitation at room temperature using a rotator. Usually, HPLC analysis showed complete consumption of the imidazolate after 24 h. The reaction mixture was diluted with imidazole buffer (0.3 M, pH = 7.0) and centrifuged. The supernatant was purified by ion exchange chromatography using a triethylammonium bicarbonate gradient elution on a Q-Sepharose FF column as described for aGTP. Removal of eluent by rotary evaporation gave 20.5 mg of a colorless oil containing 10.1 mg aGppNHp (15.9 µmol, 30 %) as determined by UV spectrometry (ε_{260nm} = 12080 $cm^{-1}M^{-1}$)[16].

HR-ESI-MS: 634. 08301 Da (theoretical mass of the charged ion 634.08233 Da, deviation: 1.06091 ppm).

4. Supplementary References

1. Muller, M.P. et al. The Legionella effector protein DrrA AMPylates the membrane traffic regulator Rab1b. *Science* **329**, 946-9 (2010).
2. Wu, Y.W. et al. A protein fluorescence amplifier: continuous fluorometric assay for rab geranylgeranyltransferase. *Chembiochem* **7**, 1859-61 (2006).
3. Oesterlin, L.K., Goody, R.S. & Itzen, A. Posttranslational modifications of Rab proteins cause effective displacement of GDP dissociation inhibitor. *Proc Natl Acad Sci U S A* **109**, 5621-6 (2012).
4. Schoebel, S., Cichy, A.L., Goody, R.S. & Itzen, A. Protein LidA from Legionella is a Rab GTPase supereffector. *Proc Natl Acad Sci U S A* **108**, 17945-50 (2011).
5. Kleywegt, G.J. & Jones, T.A. Model building and refinement practice. *Methods Enzymol* **277**, 208-30 (1997).
6. Vagin, A.A. et al. REFMAC5 dictionary: organization of prior chemical knowledge and guidelines for its use. *Acta Crystallogr D Biol Crystallogr* **60**, 2184-95 (2004).
7. Traut, T.W. Physiological concentrations of purines and pyrimidines. *Mol Cell Biochem* **140**, 1-22 (1994).
8. Wu, Y.W. et al. Membrane targeting mechanism of Rab GTPases elucidated by semisynthetic protein probes. *Nat Chem Biol* **6**, 534-40 (2010).
9. Schoebel, S., Oesterlin, L.K., Blankenfeldt, W., Goody, R.S. & Itzen, A. RabGDI displacement by DrrA from Legionella is a consequence of its guanine nucleotide exchange activity. *Mol Cell* **36**, 1060-72 (2009).
10. Gavriljuk, K. et al. Catalytic mechanism of a mammalian Rab.RabGAP complex in atomic detail. *Proc Natl Acad Sci U S A* **109**, 21348-53 (2012).
11. Alexandrov, K. et al. Characterization of the ternary complex between Rab7, REP-1 and Rab geranylgeranyl transferase. *Eur J Biochem* **265**, 160-70 (1999).
12. Muller, M.P. et al. Characterization of enzymes from Legionella pneumophila involved in reversible adenylylation of Rab1 protein. *J Biol Chem* **287**, 35036-46 (2012).
13. Cadogan, J.I.G., Roy, D.A. & Smith, D.M. An alternative to the Sandmeyer reaction. *Journal of the Chemical Society C: Organic*, 1249-1250 (1966).
14. Yoshikawa, M., Kato, T. & Takenishi, T. Studies of Phosphorylation. III. Selective Phosphorylation of Unprotected Nucleosides. *Bulletin of the Chemical Society of Japan* **42**, 3505-3508 (1969).
15. Ludwig, J. A new route to nucleoside 5'-triphosphates. *Acta Biochim Biophys Acad Sci Hung* **16**, 131-3 (1981).
16. Cavaluzzi, M.J. & Borer, P.N. Revised UV extinction coefficients for nucleoside-5'-monophosphates and unpaired DNA and RNA. *Nucleic Acids Res* **32**, e13 (2004).
